# Readability and topics of the German Health Web: Exploratory study and text analysis

**DOI:** 10.1371/journal.pone.0281582

**Published:** 2023-02-10

**Authors:** Richard Zowalla, Daniel Pfeifer, Thomas Wetter

**Affiliations:** 1 Department of Medical Informatics, Heilbronn University, Heilbronn, Germany; 2 Center for Machine Learning, Heilbronn University, Heilbronn, Germany; 3 Institute of Medical Informatics, Heidelberg University Hospital, Heidelberg, Germany; 4 Department of Biomedical Informatics and Medical Education, University of Washington, Seattle, Washington, United States of America; University of Sharjah, UNITED ARAB EMIRATES

## Abstract

**Background:**

The internet has become an increasingly important resource for health information, especially for lay people. However, the information found does not necessarily comply with the user’s health literacy level. Therefore, it is vital to (1) identify prominent information providers, (2) quantify the readability of written health information, and (3) to analyze how different types of information sources are suited for people with differing health literacy levels.

**Objective:**

In previous work, we showed the use of a focused crawler to “capture” and describe a large sample of the “German Health Web”, which we call the “Sampled German Health Web” (sGHW). It includes health-related web content of the three mostly German speaking countries Germany, Austria, and Switzerland, i.e. country-code top-level domains (ccTLDs) “.de”, “.at” and “.ch”. Based on the crawled data, we now provide a fully automated readability and vocabulary analysis of a subsample of the sGHW, an analysis of the sGHW’s graph structure covering its size, its content providers and a ratio of public to private stakeholders. In addition, we apply Latent Dirichlet Allocation (LDA) to identify topics and themes within the sGHW.

**Methods:**

Important web sites were identified by applying PageRank on the sGHW’s graph representation. LDA was used to discover topics within the top-ranked web sites. Next, a computer-based readability and vocabulary analysis was performed on each health-related web page. Flesch Reading Ease (FRE) and the 4^th^ Vienna formula (WSTF) were used to assess the readability. Vocabulary was assessed by a specifically trained Support Vector Machine classifier.

**Results:**

In total, n = 14,193,743 health-related web pages were collected during the study period of 370 days. The resulting host-aggregated web graph comprises 231,733 nodes connected via 429,530 edges (network diameter = 25; average path length = 6.804; average degree = 1.854; modularity = 0.723). Among 3000 top-ranked pages (1000 per ccTLD according to PageRank), 18.50%(555/3000) belong to web sites from governmental or public institutions, 18.03% (541/3000) from nonprofit organizations, 54.03% (1621/3000) from private organizations, 4.07% (122/3000) from news agencies, 3.87% (116/3000) from pharmaceutical companies, 0.90% (27/3000) from private bloggers, and 0.60% (18/3000) are from others. LDA identified 50 topics, which we grouped into 11 themes: “Research & Science”, “Illness & Injury”, “The State”, “Healthcare structures”, “Diet & Food”, “Medical Specialities”, “Economy”, “Food production”, “Health communication”, “Family” and “Other”. The most prevalent themes were “Research & Science” and “Illness & Injury” accounting for 21.04% and 17.92% of all topics across all ccTLDs and provider types, respectively. Our readability analysis reveals that the majority of the collected web sites is structurally difficult or very difficult to read: 84.63% (2539/3000) scored a WSTF ≥ 12, 89.70% (2691/3000) scored a FRE ≤ 49. Moreover, our vocabulary analysis shows that 44.00% (1320/3000) web sites use vocabulary that is well suited for a lay audience.

**Conclusions:**

We were able to identify major information hubs as well as topics and themes within the sGHW. Results indicate that the readability within the sGHW is low. As a consequence, patients may face barriers, even though the vocabulary used seems appropriate from a medical perspective. In future work, the authors intend to extend their analyses to identify trustworthy health information web sites.

## Introduction

### Overview

The Internet has become an increasingly important resource for health information, especially for lay people [1–7]. Web users perform online searches to obtain health information regarding diseases, diagnoses, and different treatments [1]. However, the information found does not necessarily comply with the users’ health literacy level and–consequently–might not be well understood by the respective reader. This can result in an overall poorer general health status, as well as greater barriers for the access to adequate medical care [8].

In addition, another major problem of written information is the gap between the language of medical experts and lay people. Even with a higher level of education, medical vocabulary poses problems for people reading relevant health information [9]. Moreover, the medical terms associated with the etiology of a disease tend to differ between health professionals and patients [10–12].

Health information on the web is provided by different stakeholders, each with its own set of interests [4]. Thus, the provided health information material does not necessarily reflect the needs of a (lay) health information seeker. Therefore, it is important to (1) identify information providers, (2) quantify the readability of as well as the type of vocabulary, and (3) to analyze how different types of information sources are suited for people with differing health literacy levels.

Given the great variety and vast amount of health information available on the internet, a manual or semiautomatic approach for analysis seems futile. To the best of the authors’ knowledge, there exists no study that applies machine learning methods in order to find relevant health information and that determines its readability level as well as its vocabulary level in a fully automated approach.

As a follow-up of the research conducted by Zowalla et al. in [13], this study provides a fully automated readability and vocabulary analysis of the health-related web restricted to web pages in German. We limit our study to the three predominantly German-speaking countries Germany, Austria, and Switzerland (D-A-CH) and call the sample of the “German Health Web” (GHW) acquired by our focused web crawler the “Sampled German Health Web” (sGHW). In addition, our study per country finds the 1000 top-ranked information providers in the sGHW according to PageRank and uses Latent Dirichlet Allocation (LDA) to find abstract topics as present within the sGHW.

### Related work

#### Readability of health information material

The health literacy level of an individual living in Europe was assessed within the European Health Literacy Survey. It offers an instrument with a scale ranging from 1 (lowest) to 50 (highest) and was used to compare health literacy levels in different European countries. For Germany, Zok reports an average score of 31.9 for participants, which was below the European average score (33.8) [14]. In 2016, Schaeffer et al. reported that “54.3% of [German study participants] were found to have limited health literacy” (n = 2000) [15]. For Switzerland, Bieri et al. reported, that 54% of the study participants (n = 2000) were found to have limited health literacy [16]. Pelikan et al. [17] reported, that 51.6% of the Austrian study participants (n = 1813) achieved a limited health literacy level. These findings support the need for online health information materials that meet the capabilities of their readers. Consequently, such information should be written at a sufficient readability level and (medical) specialty language should be avoided in order to reduce barriers for patients.

However, several studies found that online health information is often written and published with low readability, which reduces or even hinders understandability for its intended readers (mainly laymen) [18–27].

A recent analysis by Brütting et al. [18] about prominent web sites (n = 45) on melanoma immunotherapy written in German revealed low readability scores according to the Flesch Reading Ease Scale (FRE), which ranges from 0 to 100. A low FRE indicates an unsufficient level of readability while a high FRE indicates easy-to-read text material. In 2018, Basch et al. [19] assessed the readability of online information material related to prostate cancer. They found that the “majority of web sites had difficult readability” and concluded that a “large majority of information available on the Internet about prostate cancer will not be readable for many individuals.”

Similar studies were conducted for other diseases: Thomas et al. [20] analyzed nephrology related Wikipedia articles written in English as a resource for patient education. The overall mean FRE was 19.4, which corresponds to an unsufficient level of readability. A study by Edmunds et al. [21] assessed the readability of 160 web sites providing ophthalmic patient information and found “83% [..] as being of ‘difficult’ readability.” Tulbert et al. [22] assessed the readability of “three sources of patient-education material on the internet (WebMD.com, Wikipedia.org, and MedicineOnline.com)”. They found that “no single source of commonly used internet patient-education material demonstrates optimal features with regard to readability, length, and presence of photographic illustrations.”

In 2014, Zowalla et al. used a specifically trained Support Vector Machine (SVM) to assess the difficulty of health-related text material [28]. It was trained to distinguish between documents written for laymen and documents written for (medical) experts on the basis of 10.000 texts from various German health content providers. The resulting SVM classifier was tested against two datasets (n1 = 1202, n2 = 1200) and achived an accuracy of 0,8458 and 0,8741 respectively. Subsequently, it was applied to online health websites in the context of a Firefox browser extension in 2015 [29]. The SVM outputs a class probability using Platt Scaling [30]. This class probability is then transformed to an “expert level” expressing vocabulary-based text difficulty, which was named L.

In 2018, Zowalla and Wiesner [23] analyzed 2931 articles of the „Public Health Portal of Austria”(www.gesundheit.gov.at) using FRE, the 4^th^ Vienna formula (WSTF) and the measure L. Their analysis revealed low readability levels paired with a “moderate level of vocabulary difficulty.” In 2018, L, WSTF and FRE were also applied by Keinki et al. [24] on 51 German cancer information booklets. They report, “that the majority of the 51 booklets (92.16%) is hard to read”. In 2020, the study design was replicated by Wiesner et al. [25] for Psoriasis/Psoriatic Arthritis material written in German. They found, that “patient education materials in German require, on average, a college or university education level [..] even though the vocabulary used seems appropriate”.

McInnes and Haglund [26] entered 22 health condition terms in five different search engines and computed the readability scores of the first 10 web sites retrieved via each individual search using the Gunning Fog Index (FOG), Simple Measure of Gobbledygook score (SMOG), Flesch-Kincaid Grade (FKG) and FRE. They found, that “Websites with.gov and.nhs TLDs [top level domains] were the most readable while.edu sites were the least”. A recent study by Worrall et al. [27] used Google search to collect the first 20 web pages for searches related to the coronavirus diseases and assessed the readability using FOG, FRE, FKG and SMOG. They conclude that “only 17.2% [(n = 165)] of web pages [were] at a universally readable level.” In addition, Worrall et al. reported, that “Public Health organisations and Government organisations provided the most readable COVID-19 material, while digital media sources were significantly less readable” [27].

In addition to classic readability metrics such as FRE or WSTF, other approaches for computing the readability of (German) text material exist. In [31], vor der Brück et al. describe the readability checker DeLite, which uses 48 morphological, lexial, syntactic, and semantic indicators to assess the readability of a text written in German. A similar approach is presented by Berends and Vajjala in [32], which uses 165 custom features to assess the readability of German geography text books for secondary school. However, neither approach can easily be applied as the related source code is not publicly available. In addition, these tools are not commonly used for readability assessment of (health-related) text material.

Other studies [33–35] leveraged crowd sourcing to measure the readability of text material. In this context, crowd workers are used to judge the readability of a given text. However, such approaches require high financial resources as the related crowd workers need to be paid. The costs highly depend on the amount of text material to be reviewed, which might not be feasible for large scale analyses of text material from the web.

#### Topic modeling on health information material

Topic modeling is a well-accepted technique to discover abstract topics in unstructured text. It is often applied to clinical and/or health-related content posted on social media, online newspapers or on web sites in general [36–42].

In 2014, Paul and Dredze [36] showed, that topic models can be leveraged to infer health topics in Twitter messages. To do so, they analyzed 144 million health-related Twitter posts and discovered 13 topics, e.g. “cancer & serious illness”, “dental health”, “exercises” or “injuries & pain”, in the dataset. Another study by Liu and Yin [39] used topic modeling to analyze the abstract topics of 477,904 posts in *r/loseit* of the *reddit* community. They identified 25 topics concerning the overall theme “weight loss” such as “food and drinks”, “exercises”, or “communication”.

Another study by Muralidhara and Paul [37] leveraged topic modeling to discover the abstract health-related topics contained in 96,426 Instagram posts with hashtags related to health. Overall, they identified 47 health-related topics covering ten broad themes such as “acute illness”, “alternative medicine”, “chronic illness and pain”, or “substance use”. The most prevalent topics were related to “diet” and “exercise”.

In 2017, Melkers et al. [38] assessed the content of 89 dental blogs by using topic modeling techniques. In total, the authors found 176 abstract topics inside the data and grouped them into four leading themes: “Status/Social”, “Dental care”, “Dental practice related”, and “Other”.

Liu et al. [40] collected 642 newspaper articles related to third hand smoke and analyzed the text material by using LDA. They discovered ten topics, e.g. “cancer”, “risks of smoking”, or “air quality” and grouped them into three major themes.

In 2020, Min et al. [42] analyzed the content of 145 web sites related to “occupational accidents” by using topic modeling. They discovered 14 topics with three themes: “workers’ compensation benefits”, “illicit agreements with the employer”, and “fatal and non-fatal injuries and vulnerable workers”.

Bahng and Lee [41] analyzed posts on the social question-and-answer platform “Naver Knowledge-iN” by using LDA “to identify patients’ perceptions, concerns, and needs on hearing loss.” They found 21 topics, which “mostly correspond to sub-fields established in hearing science research”, and grouped them into five main themes such as “noise-induced hearing loss” or “sudden hearing loss”.

#### Crawling the German Health Web

In 2020, we demonstrated the suitability of a distributed focused web crawler for the acquisition of a large sample of the GHW [13]. The presented system run for 277 days and had an average harvest rate of 19.76% and the recall estimated via a seed-target approach was 0.821, which indicates, that our approach is a suitable method to acquire most health-related content found under the country-code top-level domains (ccTLDs) “.de”, “.at”, and “.ch”. The crawler uses an SVM text classifier to estimate the health relevance of a given web page. It was trained on a large data set (n = 70.048) acquired from various German content providers to distinguish between health-related and non–health-related web pages. The classifier was evaluated based on two different datasets. The first dataset (TD1) consisted of 17.514 documents and was based on a-priori class labeling, the second one (TD2) consisted of 384 real-world web pages and was annotated by using a crowd sourcing approach. Both, TD1 and TD2, had an equal class distribution. The system achieved an accuracy of 0.937 for TD1 (TD2: 0.966), precision on TD1 of 0.934 (TD2 = 0.954), and a recall of 0.944 (TD2 = 0.989). The results indicated that the presented crawler was a suitable method for acquiring a large sample of the GHW in a fully automated manner. Subsequently, we call the acquired sample of the GHW the “Sampled German Health Web” (sGHW).

This paper presents a follow-up study of the research conducted in 2020 [13]. The latter analyzes the acquired data, namely the sGHW graph and the content of health-related web pages after running the distributed focused web crawler presented in [13] for 370 days.

#### Aims of the study

In line with the methodology presented in [13], the authors decided to concentrate on health-related web pages available free of charge on the internet in the D-A-CH region that can be found under the respective ccTLDs “.de”, “.at”, and “.ch”. In this context, the aim of this study was four-fold:

1. Analyze the current situation, that is, the volume of and the information providers behind health-related web pages in the D-A-CH region.
2. Demonstrate the suitability of a fully automated approach to compute the following three aspects of the sGHW: its readability by using established readability formulas, its type of vocabulary, and the prevalent topics.
3. Quantify the level of readability of and the type of vocabulary used within the sGHW. In addition, identify the topics presented within health-related web pages in the sGHW.
4. Evaluate whether web pages offered by certain types of information providers are better suited for citizens with lower health literacy levels than others.

## Methods

### Definition of health information

In the context of this study, we define “health information” or the “health relevance” of a given web page very openly. Therefore, we include, among others, the following topics:

- Diseases and their diagnoses,
- Diagnostic procedures, therapies or treatments,
- Pharmaceutical Information (e.g., about medications),
- Homeopathy,
- Nutrition, sports and lifestyle information that is intended to lead to a “healthier” life (prevention),
- Information on health care structure (hospitals, doctor’s offices, etc),
- Information from and about self-help groups,
- Content generated by patients or users on the topic of health, e.g. in social media or internet forums.

Thus, websites considered as “health-related” do not necessarily comply with the criteria of evidence-based medicine and may have both laypersons and professionals as their target audience. Information on the health condition of animals or their treatment (veterinary medicine) is not considered as health information in the context of this study.

### Study setting

This study of health-related web pages consisted of four stages:

1. Regarding study aim 1, we used the focused web crawling system presented in [13] to collect health-related web pages and to create a health-related host-aggregated web graph. As in [13], we applied the PageRank algorithm [43] to identify important web sites in the sGHW on the aforementioned graph representation.
2. Then, one author screened the 1000 top-ranked web sites for each ccTLD by visiting the related web site in the incognito mode of a Chromium browser. In addition, the same author looked for legal information (imprint) of the web site’s owner. If a legal entity could be identified, a background check was conducted using popular search engines.
3. Based on these findings, one of the following nine categories was assigned to each web site’s information provider: Government or Public (Health) Institution (GPH), Non-Profit Organization (NPO), Private Organization or Individual Person (PO), Mainstream or Local News (M), Pharmaceutical Company (PC), Personal Blog (PB), Social Network (SN), and Other (O). The categories were defined on the basis of [13]. A detailed explanation for each category is given in S1 Appendix.
  To mitigate rater bias, the assignment was done twice with a gap of two months between each run. If there was a tie, the rater reviewed the case again and resolved it by performing an additional background check. In addition, the interrater reliability metrics percent agreement (PA) [44] and Cohen’s κ [45] were computed.
4. At the last stage, a fully automated readability and vocabulary analysis was conducted on the 1000 top-ranked web sites for each ccTLD. In addition, topic modeling was applied on the same data. The resulting topics were then paraphrased in a group discussion. These analyses were intended to answer the aims of the study 2 to 4.

### Graph analysis

Several studies have extensively analyzed the graph structure of the web [46–48]. In this context, a graph node represents a web page and an edge represents a link between two web pages. In our study, we generated a host-aggregated graph in order to reduce its computational complexity and explore its properties [49]. To do so, individual web pages are combined and represented by their parent web site (including outgoing and ingoing links). On the resulting host-aggregated sGHW graph, we applied the following metrics or algorithms:

- Average degree is the average number of edges connected to a node [50]. For a directed web graph, this is defined as the total number of edges divided by the total number of nodes.
- Modularity measures the strength of division of a graph into clusters or groups [50, 51]. Graphs with a high modularity have dense connections between the web sites within certain clusters but sparse connection to other web sites, which are contained in different clusters.
- PageRank is a centrality-based metric that allows identification of web sites (nodes) of importance inside a graph [43]. The underlying assumption is that an important graph node (web site) will receive more links from other important nodes (i.e., higher in-degree).

Other metrics such as network diameter and the average path length (i.e., the average number of clicks which will lead from one web site to another) are frequently used for graph analysis [50, 52].

### Coverage of relevant web sites

The coverage (or completeness) of our focused web crawl was evaluated by comparing the overlap to another web crawl. For this purpose, search results of the commercial search engine provider Google were used. The underlying assumption is that a (commercial) search engine provider such as Google has already indexed a large part of the web. To compute the overlap, search queries with relevant (medical) terms were sent to the application programming interface (API) of the related search engine over a period of time. Based on the results, it is then possible to determine the percentage of URLs returned by Google that are included in our focused web crawl. The related proportion is an indicator regarding the completeness of our sampled dataset.

### Web site ranking strategies

Web sites can be ranked by using different, potentially combined approaches ranging from estimating the traffic of a given website, the amount of unique visitors in a given timeframe, manual or search-engine based approaches or graph-based ranking algorithms [53]. Many ranking strategies originate from the field of search engine optimization (SEO) and aim to reproduce confidential black box ranking algorithms of (commercial) search engine providers such as Google.

In most cases, related metrics and rankings are offered by commercial third party providers such as ALEXA [54], Sistrix [55], Searchmetrics [56] or SimilarWeb [57] as part of their business. However, their methods to rank a given web site as well as influencing factors remain confidential. Obviously, this leaves an enormous gap with respect to transparency and reproducibility [53, 58].

In this study, we solely relied on PageRank [43], a clearly defined and transparent algorithm which is well established in computer science in order to assess the relevance of graph nodes. In particular, we apply PageRank to the host-aggregated graph representation of the sGHW. Therefore, our ranking is not based on any traffic estimations, popularity or visibility indices measured by third party providers. Moreover, it is not influenced by commercial interests and can easily be reproduced by other researchers. It provides a ranking of the sGHW based on its link structure as collected by our focused web crawler.

### Readability analysis

#### Definition

Readability describes the properties of written text with respect to the readers’ understanding of a document [59, 60]. It depends on the complexity of a text’s structure, the sentence structure and the vocabulary used.

#### Flesch reading ease scale

The FRE is a well-established readability metric for the English language [61]. FRE relies on the average sentence length (ASL) and the average number of syllables per word (ASW). FRE assumes that short words or sentences are usually easier to understand than longer ones. We applied the modified FRE scale by Toni Amstad [62] for the German language. It is defined as follows:

FRE=180-ASL-(58.5×ASW)

#### Vienna formula

In contrast to the FRE, the Vienna formula (WSTF) was originally developed for the German language by Bamberger and Vanacek [63]. They derived different versions of the Vienna formula for prose and non-fictional text. Typically, the 4^th^ WSTF is used for text analysis. It relies on the average sentence length (ASL) and on the proportion of (complex) words with three or more syllables (MS):

4thWSTF=0.2656×ASL+0.2744×MS-1.6939

#### Vocabulary-based text difficulty

The German language makes use of many compound words (e.g. “Halsschmerzen”, “Magen-Darm-Erkrankung”, “Zuckerkrankheit”). These terms are quite layman friendly (for an average patient) but are very lengthy. Consequently, average word length or syllable counts are not a good indicator to decide if a given word is easily comprehensible (that means, if it can be easily understood by people with a grade level of 6–7).

Machine learning techniques can be used to compensate for the limitations of established sentence-based readability measures such as FRE scale or WSTF [28, 64].

To quantify the vocabulary-based text difficulty (i.e., the “expert-centricity” of a given text), we defined the measure L ∈ [1, .., 10] similar to [23–25, 29], which leverages the SVM classifier of [28] as described in “Related Work”. Before using this pretrained classifier to assess the vocabulary-based difficulty of medical text material, several preprocessing steps are necessary [65]. As a first step, text material is cleaned from syntactic markup (i.e. boilerplate code, HTML tags). Next, each text is tokanized (i.e. split into single word fragments) and each character is converted to lower case (case folding). Stop words are removed (e.g. “the”, “and”, “it”) as they do not influence the difficulty of a text. Next, stemming techniques are applied in order to map tokens to their stems and reduce morphological variations of words (e.g. “goes” becomes “go”). Finally, the text content of a document is transformed into a document vector based on previously selected features from [28]. For each text, the SVM classifier outputs a class probability using Platt Scaling [30]. The class probability is then transformed to the value L, which expresses vocabulary-based text difficulty.

Low values of L indicate a very easy text written for the elementary level or elementary school; a value of 3–4 corresponds to an easy text (intermediate level / junior high school), a value of 4–5 to a moderate text (laymen with medical educational background), a value of 5–6 to a difficult text, a value of 7–8 to a very expert-centric text and a value of > 8 indicates that an academic (medical) background knowledge or working experience in the medical domain is required. The procedure and the related processing steps are described in detail in [29].

### Topic modeling

In this study, we applied topic modeling to identify themes and topics within the GWH. Specifically, we used LDA to identify the main topics of the three times 1000 top-ranked web sites [66]. Since LDA is an unsupervised algorithm, we relied on perplexity to determine the optimal number of topics [66]. To do so, we trained LDA models using Gibbs Sampling [67] with 3000 iterations for 1 to 90 topics (with a step size of 10) on the full dataset of the three times 1000 top-ranked web sites consisting of 3,746,055 web pages. To mitigate word sparsity, we conducted stemming and removed words with little to no analytical value (e.g., “der” (article), “und” (conjunction), “jetzt” (particle)). In addition, only words with a minimum frequency of 200 were kept in the text corpus.

To estimate LDA’s hyper parameters (named α and β), we applied a method from Asuncion et al. [68] which is based on Minka [69] and an EM procedure nesting the actual Gibb’s sampling algorithm. Thus, the approach determines optimized hyper parameters as part of the topic inference. Moreover, we relied on Wallach et al. (Equation 7) [70] in order to assess the prevalence of topics in web pages as described in [71] (Section 3.4). To describe the statistical dispersion of the topic distribution, we used the Gini coefficient [72].

The preprocessing steps and software libraries used to conduct this analysis are described in more detail in Section “Computational Processing & System Environment“.

Each topic consists of a set of keywords and was visualized using word clouds. The word clouds were subsequently labeled by eight volunteers with different backgrounds including “Medical Informatics”, “Health Economics”, “Physics”, “Social Economics”, “Marketing”, and “Electrical Engineering”: A spread sheet document containing the word clouds to be labeled was provided along with instructions to each volunteer (see S2 Appendix). The results were then aggregated by one of the authors and given to two other volunteers (“Medical Informatics” and “Civil Engineering”), who conducted the final paraphrazing for each topic in a group discussion. Summarization into themes was conducted via a group discussion among two of the authors.

### Graph analysis

The graph database Neo4j, version 4.1.1, was used to store the host-aggregated web graph, which was generated by the focused crawler. The Neo4j graph algorithm plugins were used to compute PageRank and related metrics on an Ubuntu 20.04 LTS 64-bit server.

### Statistical analysis

The statistics software R (The R Foundation for Statistical Computing), version 3.6.3 (February 29, 2020), on an Ubuntu 20.04 LTS 64-bit computer was used to compute PA, Cohen’s κ and the Pearson correlation coefficient (PCC).

### Computational processing & system environment

#### Readability analysis

Given the results of our previous study [13], it became obvious that sequential processing of the huge amount of crawled data would take too much time and resources. For this reason, a parallel and distributed system architecture is necessary to process the crawled data efficiently. There are several frameworks that allow for such distributed processing; in this study, we relied on the Apache Storm framework [73]–a software development kit for building scalable computation systems in Java.

**Fig 1:**
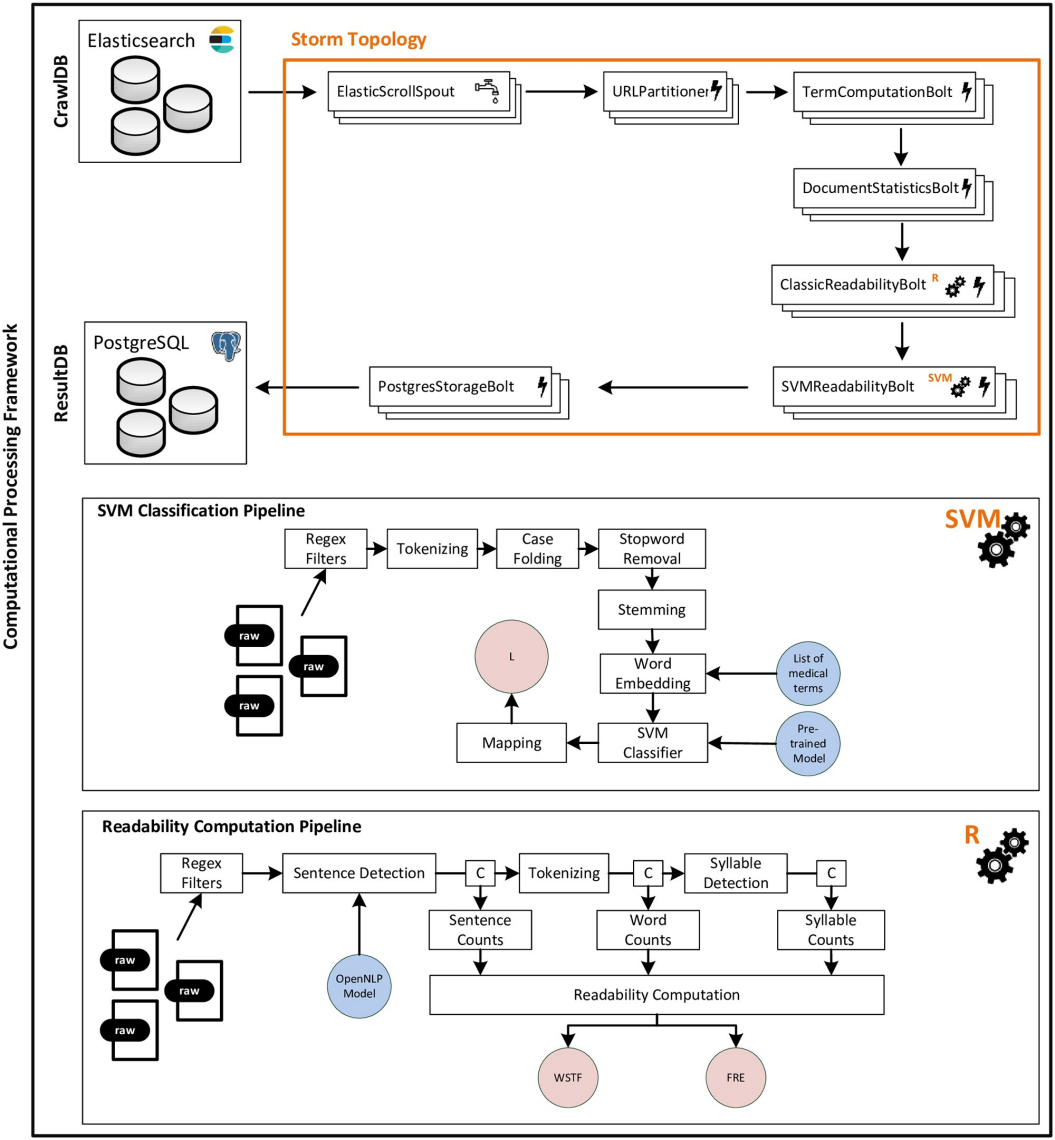
Workflow and architecture of the computational processing framework. Spouts (tap symbol) emit data (here: web pages), bolts (lightning symbol) process data (i.e. term statistics, readability metrics, vocabulary-based text difficulty, storing results). SVM: Support Vector Machine, R: Readability Metrics.

Fig 1 depicts the architecture of our distributed text analysis framework. A set of spouts emit yet unprocessed URLs along with their underlying text material (as tuples) from the crawl database. The tuples are assigned to cluster nodes (based on their hostname) and directed to text analysis components. First, the raw text material is tokenized (i.e., split into single word fragments) and transformed into a bag of words, which is added to the given tuple. Next, several statistical measures such as syllable counts, (complex) word counts, or character counts are computed.

Each tuple is then processed to compute the readability measures FRE and WSTF. To do so (see lower part of Fig 1 “gear icon” marked with the label “R”), the tuple’s full text is fed to a natural language processing (NLP) pipeline. Regular expression filters sanitize the input and remove disturbance artifacts (e.g., different hyphen encoding schemes). Finally, the aforementioned readability metrics are computed. For sentence detection, we rely on the Apache OpenNLP library [74] and its sentence model for the German language [75]. Liang’s hyphenation algorithm is used to estimate syllable counts [76].

Next, the tuple is processed to gauge the vocabulary-based text difficulty (see lower part of Fig 1, “gear icon” marked with the label “SVM”). Several pre-processing steps are necessary to apply the pre-trained classifier to our text material [28, 65]: As a first step, regular expression (regex) filters are applied in a similar manner as for FRE and WSTF. Second, a text is tokenized, converted to lower-case and stop words are removed. The latter is important as stop words do not influence the difficulty of a text. Third, the remaining tokens are reduced to their stems (e.g., goes becomes go) in order to limit linguistic variations by means of Porter’s Snowball Stemmer [77].

Each text is transformed into a bag of words representation (document vector) based on a broad list of previously selected terms from the medical domain as such terms greatly influence the vocabulary-based difficulty of a text. Each document vector is then fed into the classifier and the related output is mapped to the vocabulary measure *L*. Finally, each enriched tuple is stored in a PostgreSQL (v10.15) database for subsequent analysis.

The computing cluster consists of 22 virtual machines running on Ubuntu 18.04 LTS 64bit. Two physical servers (each equipped with two Intel Xeon E5-2689 and 256GB of memory) of a Cisco unified computing system provide the computational resources and run as a virtualization platform to allow shared resource allocation. The analysis was conducted between August 6 and August 30, 2020.

#### Topic modeling

**Fig 2:**
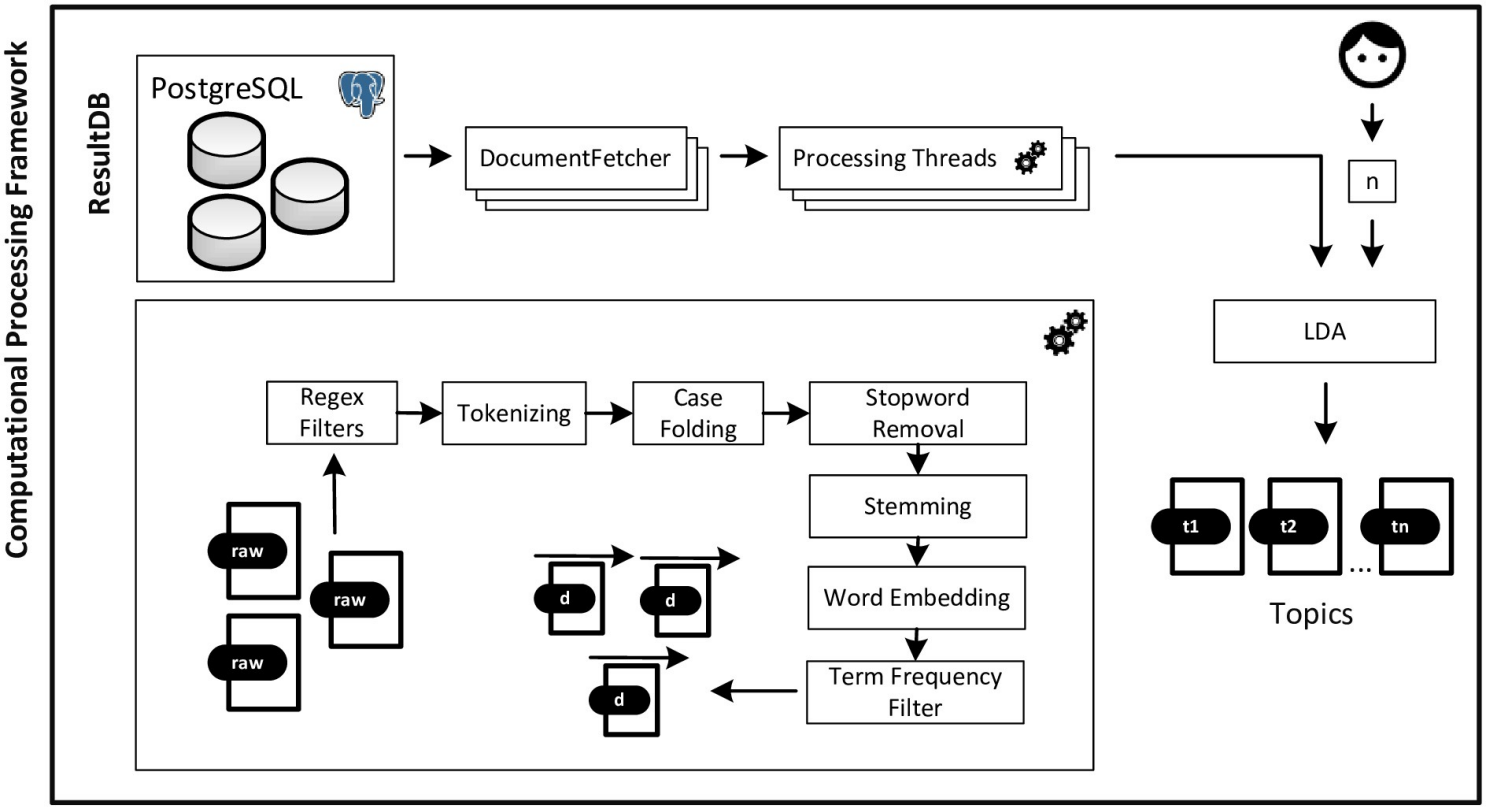
Workflow of the processing steps and software components for topic modeling: (1) text material is retrieved from a central relational database; (2) several processing threads perform a collection of pre-processing tasks; (3) LDA is applied to the resulting document vectors. The software takes raw text material as an input and outputs *n* topics. The *n* is a user-defined input parameter to LDA.

Fig 2 depicts the architecture of our analysis framework to conduct topic modeling using LDA.

As a first step, the bag of words representation of each web page is fetched by multiple threads from the PostgreSQL database containing the pre-processed web pages. If a corresponding web page had not yet been handled via the readability analysis, pre-processing steps are conducted in the same way as for the Classification pipeline from Section “Readability Analysis”. As an additional step, terms are filtered based on their minimum frequency within the document collection. Next, LDA is applied to the given document collection. We relied on the LDA implementation contained in the Topic Grouper framework by Pfeifer and Leidner [78].

The LDA-procedure and analysis to determine a reasonable number “*n*” of topics using the perplexity score (see “Methods” section) was conducted on a bare-metal server (equipped with two Intel Xeon E5-2630 v4 and 384 GB of memory) running Ubuntu 18.04 LTS with Java 11.0.9 between November 5 and December 30, 2020.

## Results

### Graph analysis

The focused web crawling system [13] ran from May 27, 2019 to May 31, 2020 and collected 14,193,743 health-related web pages. The resulting host-aggregated web graph of the sGHW comprises 231,733 nodes (web sites) connected via 429,530 edges (links between web sites).

A total of 82.63% (191,479/231,733) of the web sites belong to the ccTLD “.de”; 7.89% (18,272/231,733) to”.at”, and 9.48% (21,976/231,733) to “.ch”. The graph has a network diameter of 25. The average path length is 6.804. The average degree is 1.854. Modularity was computed to be 0.717.

**Fig 3:**
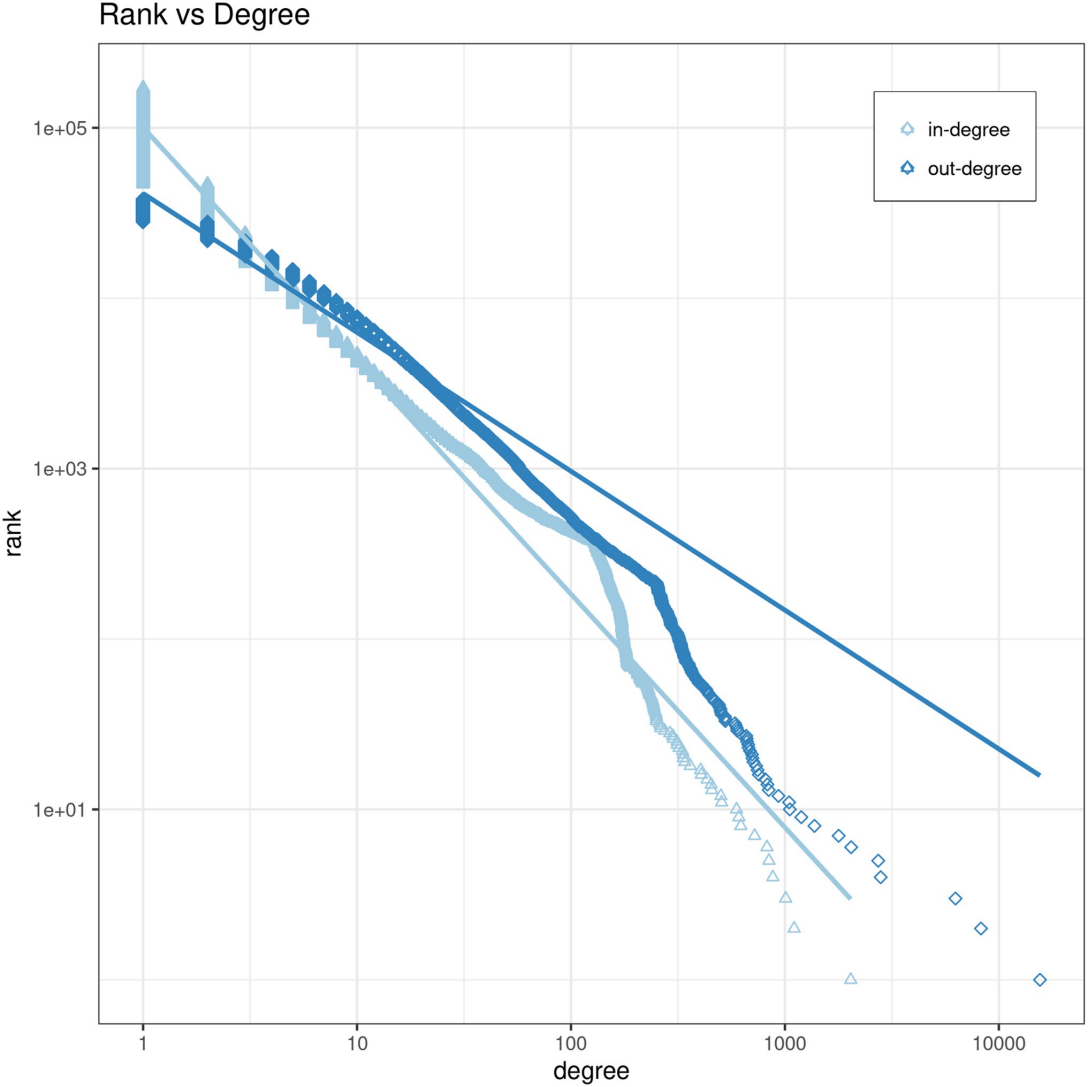
Size-rank plot of degree distribution of the host-aggregated sGHW graph.

Fig 3 depicts the size-rank plot of the degree distribution of the host-aggregated sGHW graph. In- and out-degree represent the number of hyperlinks to or from all web pages that belong to an individual host. From what we can see visually, there is a concavity, indicating that the distribution does not follow a power law. This is in line with the results by Meusel et al. in [48], who conducted a similar analysis for a host-aggegated graph of an unfocused web crawl.

As the ccTLD “.de” has the highest share within the graph, a global ranking according to PageRank would be dominated by “.de” web sites. For this reason, we used the 1000 top-ranked web sites according to PageRank in the subsequent analyses for each ccTLD separately.

### Coverage of relevant web sites

To measure the coverage of our focused web crawl, we computed the overlap of our data against the commercial search engine provider Google. For this purpose, term-based search queries were sent to a Google Search Engine configured for the ccTLDs “.de”, “.at”, and “.ch” over a period of 306 days (September 16, 2020 to July 19, 2021).

The search queries were based on the then most common diseases in Germany [79] and included the following terms: “Coronary heart disease”, “Back pain”, “Lung cancer”, “Chronic obstructive pulmonary disease COPD”, “Alzheimer’s disease”, “Falls”, “Diabetes”, “Stroke”, “Migraine headache” and “Neck pain”. In addition, search queries for ten randomly selected rare diseases [80] with the following terms were used: “Cystic Fibrosis”, “Narcolepsy”, “Gaucher disease”, “Acrodermatitis”, “Munchhausen-by-proxy syndrome”, “Niemann-Pick disease”, “Multiple endocrine neoplasia”, “Huntington’s disease”, “Creutzfeldt-Jakob syndrome”, and “Asperger syndrome”.

A total of 4,093 web sites for the most common diseases and 2,736 for the random selection of rare diseases were returned by Google. Our focused web crawl covered a total of 3,519/4,093 (85.98%) of the most web sites for common diseases and 2,425/2,736 (88.63%) of the web sites for rare diseases. In summary, the web crawl contained 5,944/6,829 (87.04%) of the web sites returned by Google.

This suggests that we obtained a high coverage of health-related German web sites as our results parallel the coverage of a very comprehensive commercial web crawler.

### Ranking of web sites

The most important host-aggregated URLs (according to PageRank) were categorized according to the categories introduced in Section “Study Setting”. The raters achieved a PA of 0.879 and a Cohen’s κ of 0.797. According to Landis and Koch [81], these κ values correspond to a “substantial agreement”. In 10.82% (364/3000) of the cases, no majority vote was achieved. Such cases were subsequently cleared following the procedure described in “Study setting”. The category “Social Network” was not selected, as no social network was contained in the 1000 top-ranked web sites for each ccTLD.

Table 1 lists the 25 top-ranked web sites according to PageRank with their respective information provider for “.de”. In total, 214 out of 1000 (21.40%) are published by governmental or public (health) institutions (GPH), 23.70% (237/1000) are published by non-profit organizations (NPO) and 43.50% (435/1000) by private organizations or individual persons (PO), i.e. web sites of medical professionals or related businesses. 62 out of 1000 (6.20%) are published by mainstream or local news agencies (M), 39 out of 1000 (3.90%) by pharmaceutical companies (PC) and 0.80% (8/1000) originated from private or personal blogs (PB). The category “Other” was given to 5 out of 1000 web sites (0.50%).

**Table 1:** Domains of 25 top-ranked web sites for ccTLD “.de” with their respective information provider according to PageRank.

Rank	Domain	Information Provider	Type
1	www.jameda.de	jameda GmbH	PO
2	www.google.de	Google LLC	O
3	www.gesetze-im-internet.de	Bundesministerium der Justiz und für Verbraucherschutz	GPH
4	www.rki.de	Robert Koch Institute	GPH
5	www.spiegel.de	Spiegel-Verlag Rudolf Augstein GmbH & Co. KG	M
6	www.aerzteblatt.de	Deutscher Ärzte-Verlag GmbH	GPH
7	www.bmbf.de	Bundesministerium für Bildung und Forschung	GPH
8	www.bundesgesundheitsministerium.de	Bundesministerium für Gesundheit	GPH
9	www.charite.de	Charité –Universitätsmedizin Berlin	GPH
10	www.aerztezeitung.de	Springer Medizin Verlag GmbH	PO
11	www.dge.de	Deutsche Gesellschaft für Ernährung	NPO
12	www.zeit.de	Zeitverlag Gerd Bucerius GmbH & Co. KG	M
13	www.g-ba.de	Gemeinsamer Bundesausschuss	GPH
14	www.welt.de	Axel Springer SE	M
15	www.apotheken-umschau.de	Wort & Bild Verlag	PO
16	www.sueddeutsche.de	Süddeutsche Zeitung GmbH	M
17	www.focus.de	Hubert Burda Media	M
18	www.bzga.de	Bundeszentrale für gesundheitliche Aufklärung	GPH
19	www.test.de	Stiftung Warentest	NPO
20	www.fraunhofer.de	Fraunhofer-Gesellschaft zur Förderung der angewandten Forschung e. V.	GPH
21	www.dimdi.de	Deutsches Institut für Medizinische Dokumentation und Information	GPH
22	www.osteopathie.de	Verband der Osteopathen Deutschland e.V.	NPO
23	www.ndr.de	Norddeutscher Rundfunk	M
24	www.bloggerei.de	Individual Person	PB
25	www.bfarm.de	Bundesinstitut für Arzneimittel und Medizinprodukte	GPH

Information provider types: GPH: Government, Public Institution or Public Health, NPO: Non-Profit Organization, PO: Private Organization, M: Mainstream or Local News:, PC: Pharmaceutical Company, PB: Private Blog, Other: O.

Table 2 lists the 25 top-ranked web sites according to PageRank with their respective information provider for “.at”. In total, 145 out of 1000 (14.50%) are published by GPH, 14.70% (147/1000) are published by NPO and 60.30% (603/1000) by PO. 40 out of 1000 (4.00%) are published by M, 46 out of 1000 (4.60%) by PC and 1.20% (12/1000) originated from PB. The category “Other” was given to 7 out of 1000 web sites (0.70%).

**Table 2:** Domains of 25 top-ranked web sites for ccTLD “.at” with their respective information provider according to PageRank.

Rank	Domain	Information Provider	Type
1	www.google.at	Google LCC	O
2	derstandard.at	Standard Verlagsgesellschaft m. b. H.	M
3	www.meduniwien.ac.at	Universität Wien	GPH
4	www.gesundheit.gv.at	Bundesministerium für Arbeit, Soziales, Gesundheit und Konsumentenschutz	GPH
5	www.femmestyle.at	Schönheitschirurgie femmestyle	PO
6	www.sozialministerium.at	Bundesministerium für Arbeit, Soziales, Gesundheit und Konsumentenschutz	GPH
7	www.ots.at	APA-OTS Originaltext-Service GmbH	PO
8	www.lknoe.at	NÖ Landesgesundheitsagentur	GPH
9	www.gettyimages.at	Getty Images (Austria) GmbH	PO
10	www.netdoktor.at	netdoktor GmbH	PO
11	www.naturavetal.at	Naturavetal GmbH & Co. KG	PO
12	www.ages.at	Österreichische Agentur für Gesundheit und Ernährungssicherheit GmbH	GPH
13	kurier.at	Kurier Zeitungsverlag und Druckerei GmbH	M
14	www.aerztekammer.at	Österreichische Ärztekammer	GPH
15	www.gaviscon.at	Reckitt Benckiser	PC
16	www.brustvergroesserung-leicht.at	Individual Person	PO
17	www.sam-pharma.at	Pharma Handel GmbH	PC
18	www.uibk.ac.at	University of Innsbruck	GPH
19	www.lehrlingstraining.at	il Aus- und Weiterbildung GmbH	PO
20	www.oesterreich-fonds.at	Nationalstiftung für Forschung, Technologie und Entwicklung	GPH
21	www.tg-steiermark.at	TG Therapeutische Gemeinschaft Betriebs GmbH	NPO
22	beta.tg-steiermark.at	TG Therapeutische Gemeinschaft Betriebs GmbH	NPO
23	www.therapie.impuls-fs.at	Institut für medizinisch-physiotherapeutische Untersuchung, Lehre und Schulung	PO
24	www.ausbildungen.impuls-fs.at	Institut für medizinisch-physiotherapeutische Untersuchung, Lehre und Schulung	PO
25	www.crystal-kolloiden.at	Crystal Colloidals B.V.	O

Information provider types: GPH: Government, Public Institution or Public Health, NPO: Non-Profit Organization, PO: Private Organization, M: Mainstream or Local News:, PC: Pharmaceutical Company, PB: Private Blog, Other: O.

Table 3 lists the 25 top-ranked web sites according to PageRank with their respective information provider for “.ch”. In total, 196 out of 1000 (19.60%) are published by GPH, 15.70% (157/1000) are published by NPO and 58.30% (583/1000) by PO. 20 out of 1000 (2.00%) are published by M, 31 out of 1000 (3.10%) by PC and 0.70% (7/1000) originated from PB. The category “Other” was assigned to 6 out of 1000 web sites (0.60%).

**Table 3:** Domains of 25 top-ranked web sites for ccTLD “.ch” with their respective information provider according to PageRank.

Rank	Domain	Information Provider	Type
1	www.uzh.ch	Universität Zurich	GPH
2	www.admin.ch	Bundesrat	GPH
3	www.usz.ch	Universitätsspital Zürich	GPH
4	www.srf.ch	Schweizer Radio und Fernsehen	M
5	www.nzz.ch	Neue Zürcher Zeitung	M
6	www.swissuniversities.ch	Rektorenkonferenz der schweizerischen Hochschulen	GPH
7	www.femmestyle.ch	Schönheitschirurgie femmestyle	PO
8	www.unibe.ch	Universität Bern	GPH
9	www.krebsliga.ch	Krebsliga Schweiz	NPO
10	www.blv.admin.ch	Bundesamt für Lebensmittelsicherheit und Veterinärwesen	GPH
11	www.zewo.ch	Stiftung Zewo	NPO
12	www.fmh.ch	Berufsverband der Schweizer Ärztinnen und Ärzte	NPO
13	www.gettyimages.ch	Getty Images (Switzerland) SA	PO
14	www.tagesanzeiger.ch	Tamedia	M
15	www.hirslanden.ch	Hirslanden AG	PO
16	www.psychologie.ch	Föderation der Schweizer Psychologinnen und Psychologen	NPO
17	www.swissmedic.ch	Swissmedic, das Schweizerische Heilmittelinstitut	GPH
18	www.naturavetal.ch	Naturavetal GmbH & Co. KG	PO
19	www.emr.ch	Eskamed AG	PO
20	www.pancreas-help.ch	Schweizer Selbsthilfegruppe Pankreaserkrankungen	NPO
21	www.mutterglueck.ch	Unknown (web site was suspended)	O
22	archiv.ever.ch	Individual Person	PO
23	astroschmid.ch	Individual Person	PO
24	www.hon.ch	Health On the Net Foundation	NPO
25	www.association-osteo-swiss.ch	Schweizerischer Verband der Osteopathen	NPO

Information provider types: GPH: Government, Public Institution or Public Health, NPO: Non-Profit Organization, PO: Private Organization, M: Mainstream or Local News:, PC: Pharmaceutical Company, PB: Private Blog, Other: O.

Overall, 555 out of 3000 (18.50%) were published by GPH, 18.03% (541/3000) by NPO, 54.03% (1,621/3000) by PO, 4.07% (122/3000) by Ms, 3.87% (116/3000) by PC and 0.90% (27/3000) by PB. The category “Other” was given to 18 out of 3000 web sites (0.60%).

S3 Appendix provides a full listing of the 1000 top-ranked web sites for each ccTLD.

### Dataset characteristics

Overall, the web pages from 2720 of the top ranked web sites were included for readability and vocabulary assessment. These web pages account for 26.39% (3,746,055/14,193,743) of the initially crawled dataset. In this sample, 75.1% (2,813,953/3,746,055) originated from the ccTLD “.de”, 9.2% (344,828/3,746,055) from “.at”, and 15.7% (587,274/3,746,055) from “.ch”.

The average number of web pages per web site ranged from 1–304,420 (mean 1375.7; median 24; SD 10,570.7). The average number of sentences per web site ranged from 1–2,836 (mean 51.182; median 29.9; SD 107.7) and the average number of words from 17–21,865 (mean 852.7; median 504.7; SD 1277.3). Complex words, i.e. ≥3 syllables, ranged from 4–10,429 (mean 307.6; median 176; SD 483.3).

A complete listing for each web site with data on the number of sentences, words, complex words, and syllables is given in S3 Appendix. 280 out of the 3000 top-ranked web sites could not be analyzed as (a) the related web pages were either not visited or not stored by our focused crawler, (b) text material could not be extracted, or (c) was too short for further analyses.

### Readability analysis

All web sites were analyzed according to the readability metrics FRE, WSTF and L, as outlined in the Methods section. The applied metrics FRE, WSTF and L are based on different scales. For a more accessible presention, we mapped the values of each scale to five classes in order to note text difficulty across the metrics in a uniform way. We applied the same mapping as presented by Wiesner et al. [25]. The mapping for each metric is given in Table 4.

**Table 4:** Mapping readability and vocabulary scales to corresponding classes as follows: VE very easy; E easy; M moderate; D difficult; VD very difficult according to Wiesner et al. [25].

Difficulty	FRE ∈ [0, 100]	WSTF ∈ [4, 15]	L ∈ [1, 10]	Class
Very difficult to read	[0–29]	14, 15	9, 10	VD
Difficult to read	[30–49]	12, 13	7, 8	D
Fairly difficult to read	[50–59]	10, 11	6	D
Average readability	[60–69]	8, 9	5	M
Fairly easy to read	[70–79]	7	4	E
Easy to read	[80–89]	5, 6	3	E
Very easy to read	[90–100]	4	1, 2	VE

The class distribution for FRE, WSTF and L, for each information provider type, is given in S4 Appendix. For the ccTLD “.de”, the web site with the lowest readability was “www.uksh.de” (n = 168,185) with an FRE value of 0.147 (SD = 2.105) and a WSTF of 14.936 (SD = 0.923). This corresponds to VD (very difficult to read). For the ccTLD “.at”, the lowest readability was computed for “www.mycare.at” (n = 1398) with an FRE value of 0.025 (SD = 0.330) and a WSTF of 15 (SD = 0) (VD). “www.implantat-berater.ch” (n = 251) had the lowest readability in “.ch” with FRE = 0.091 (SD = 0.827) and WSTF = 14.998 (SD = 0.0152) (VD). For the ccTLD “.ch”, the best readable web sites in all three countries were offered by web sites for which the focused crawler only collected a low amount of web pages (n < 10) (see S3 Appendix).

**Fig 4:**
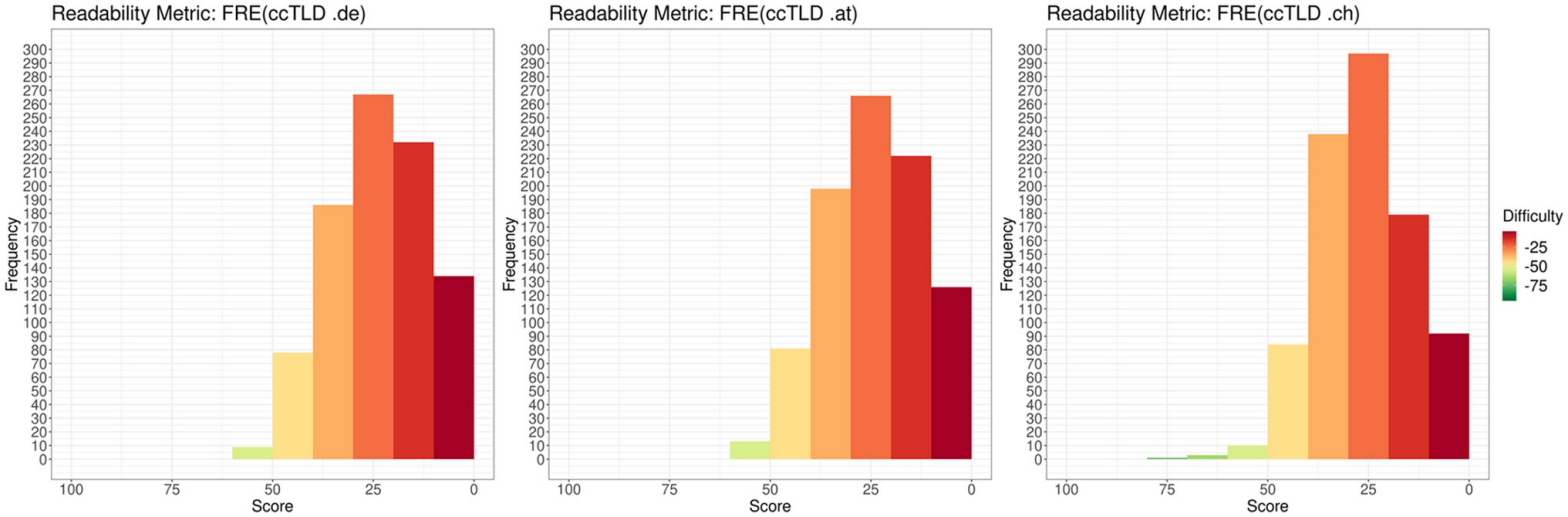
Distribution of readability values on the Flesch Reading Ease scale for each ccTLD (“.de”, “.at”, “.ch”). Difficulty indicated by color, with dark green as the highest readability (90–100) and dark red as the lowest readability (0–10). Note: For consistency reasons, the x axis is reverted and ranges from 100 to 0.

**Fig 5:**
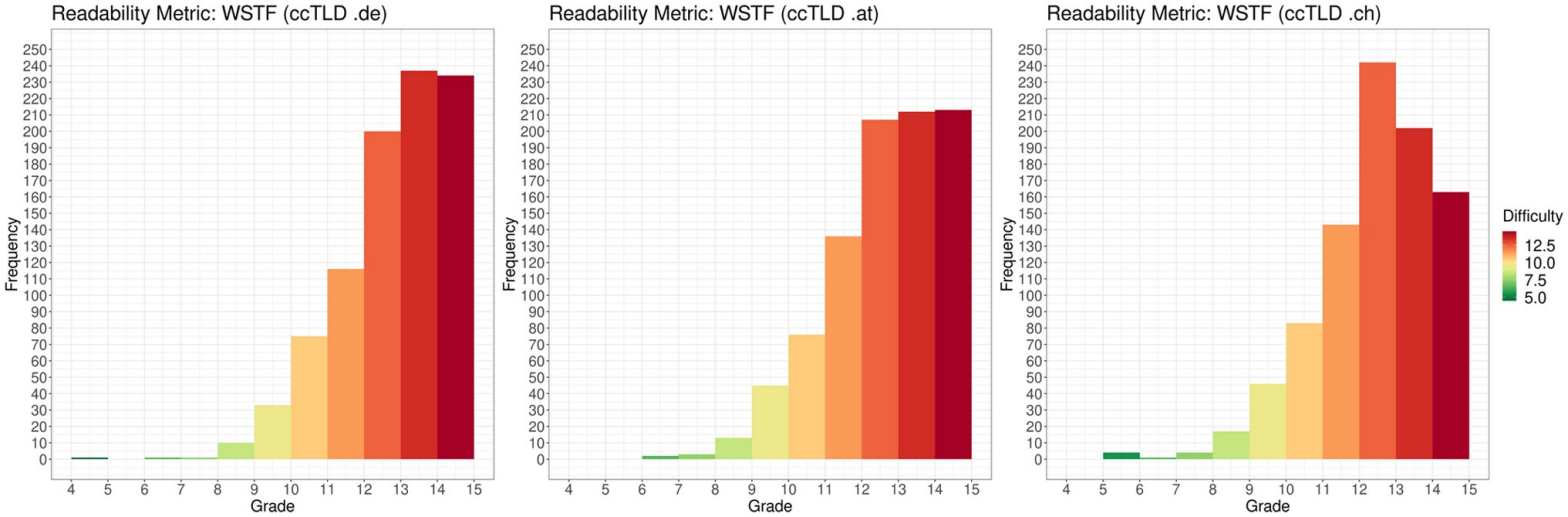
Distribution of readability values on the Vienna formula scale for each ccTLD (“.de”, “.at”, “.ch”). Difficulty is indicated by color, with dark green as the highest readability (4–5) and dark red as the lowest readability (14–15).

According to FRE, most web sites (90.533%; 2,716/3000) are difficult (D) or very difficult (VD) to read. This corresponds to the WSTF scores for which 2,539/3000 (84.633%) web sites are difficult or very difficult to read. The distributions for each ccTLD are depicted in Fig 4 (FRE) and Fig 5 (WSTF)

Regarding the vocabulary-based difficulty, a total of 568/3000 (18.93%) web sites had an L ≥9 and are thus only suitable for an academic readership. 829 out of 3000 (27.63%) web sites achieved a score ≤4 (VE+E) and are therefore suitable for a lay audience. For the remaining web sites (44.07%, 1322/3000), a score between >4 and <9 corresponds to a level suitable for persons with medical knowledge or a strong medical background.

**Fig 6:**
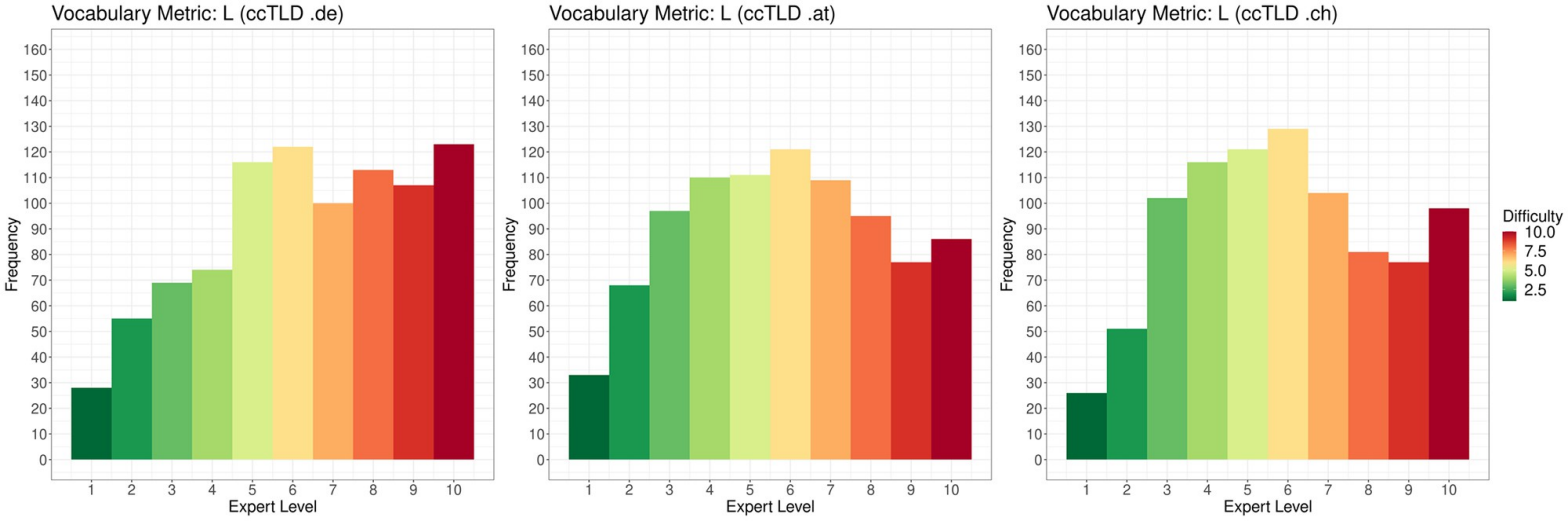
Distribution of achieved vocabulary values on the SVM classification scale L for each ccTLD (“.de”, “.at”, “.ch”). Difficulty is indicated by color with dark green as the most layman friendly (1) and dark red as the highest expert level required (10). SVM: support vector machine.

The web sites of the ccTLD “.at” scored the lowest vocabulary measure with L = 5.796 (SD = 2.543), followed by L = 5.885 (SD = 2.499) for web sites under the ccTLD “.ch”. Web sites under the ccTLD “.de” scored the highest vocabulary measure with L = 6.340 (SD = 2.572). The distribution of the classification results over all web sites is depicted in Fig 6. In this context, 281 out of the 3000 top-ranked web sites could not be analyzed for reasons explained in the “Readability Analysis” section.

**Fig 7:**
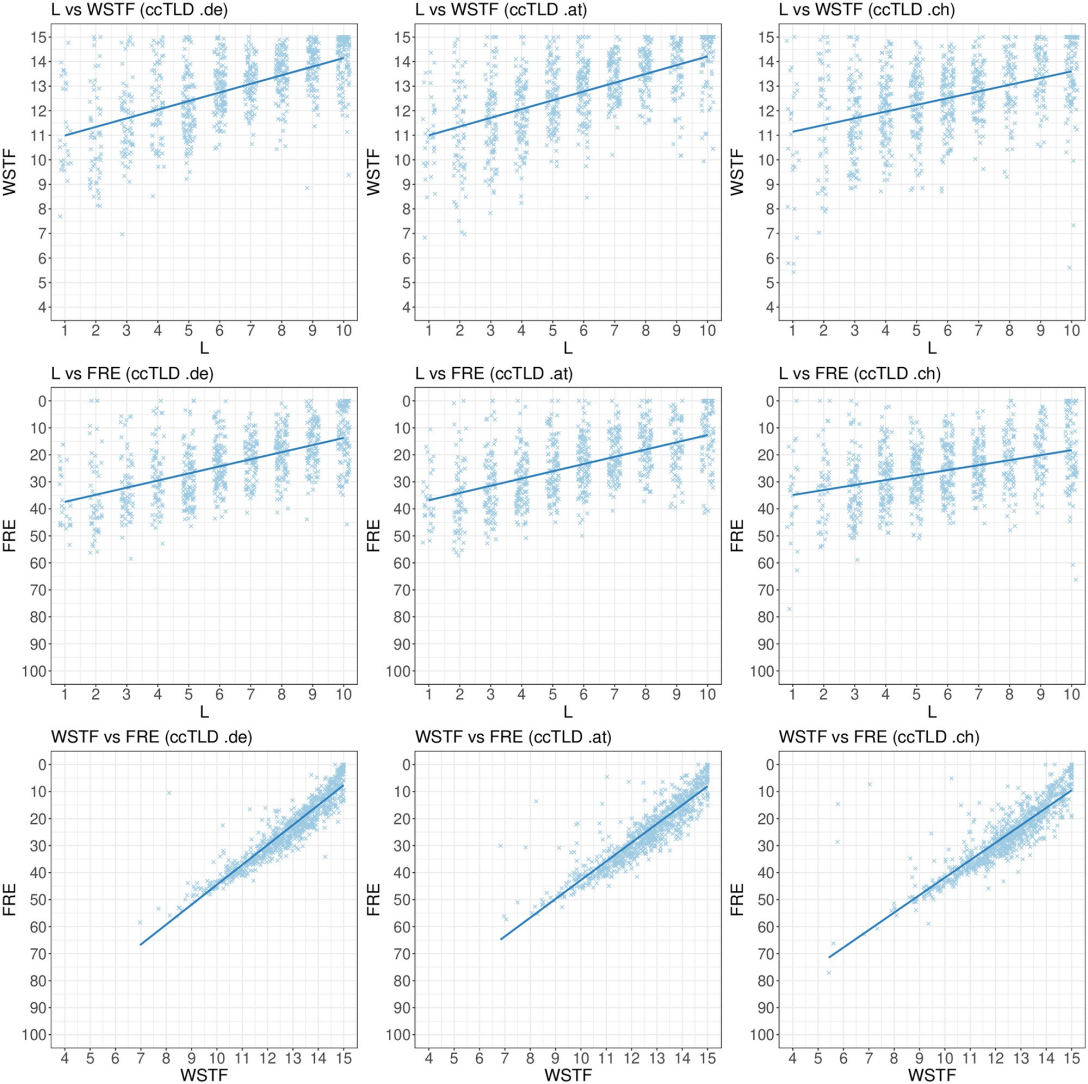
Scatter plot of the distributions for FRE, WSTF and L for each ccTLD.

Fig 7 shows a scatter plot of the distributions of FRE, WSTF and L for each ccTLD. The scatter plots indicate a correlation between FRE vs WSTF, WSTF vs L, and FRE vs L. This is confirmed by the related PCCs: PCC_de_(L, WSTF) = 0.5906, PCC_de_(L, FRE) = 0.5601, PCC_de_(WSTF, FRE) = 0.9333; PCC_at_(L, WSTF) = 0.5692, PCC_at_(L, FRE) = 0.5541, PCC_at_(WSTF, FRE) = 0.9128; PCC_ch_(L, WSTF) = 0.4234, PCC_ch_(L, FRE) = 0.3813, PCC_ch_(WSTF, FRE) = 0.8748. As one can see, WSTF and FRE are highly correlated, and therefore function as almost interchangeable measures to characterize sentence complexity. Also, high vocabulary difficulty moderately correlates with sentence complexity.

### Topic modeling

**Fig 8:**
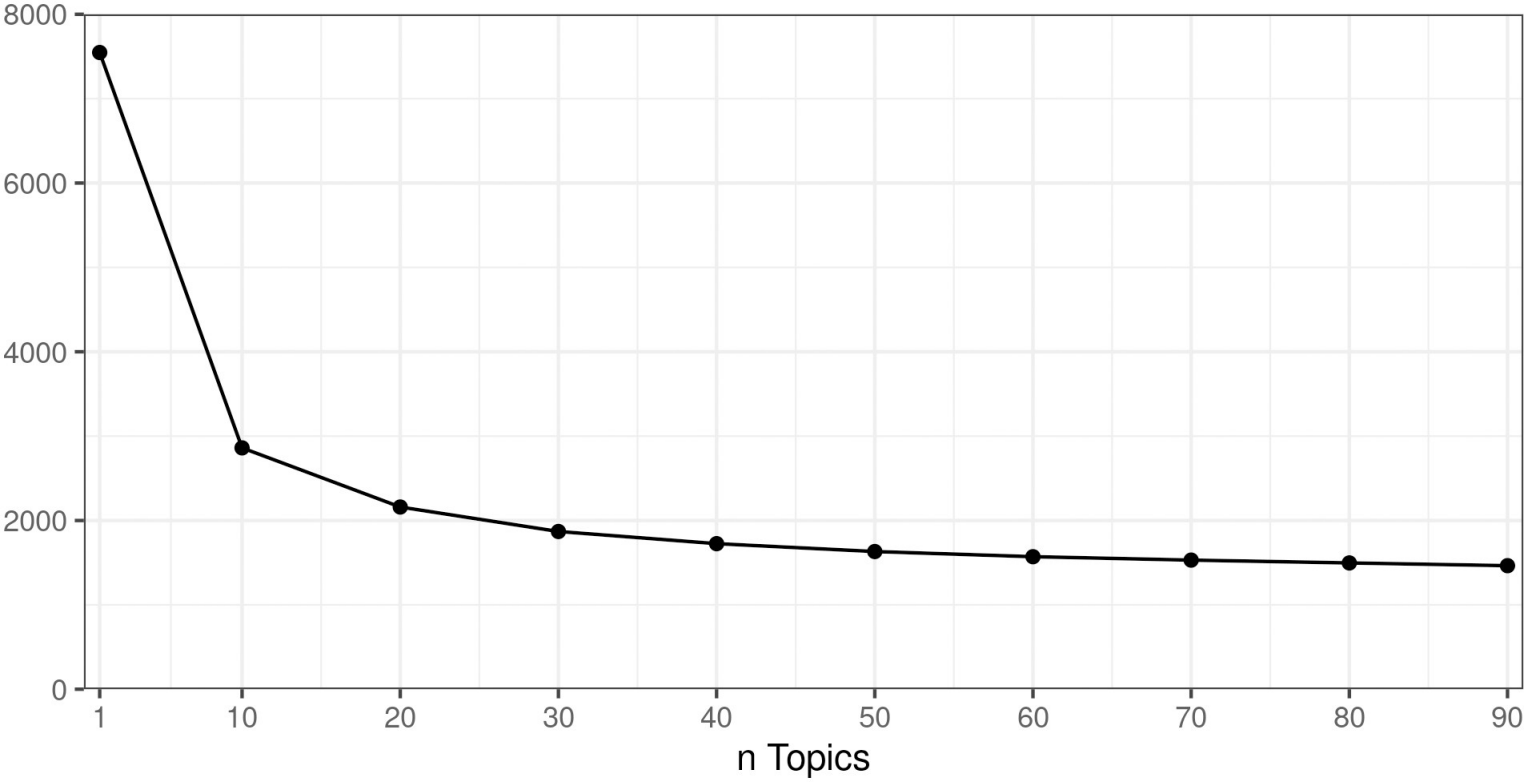
Perplexity score per number of topics for those 3,747,055 health-related web pages that belong to the three times 1000 top-ranked web sites from the sGHW.

In order to determine a suitable number of topics, we performed LDA topic modeling with a varying topic number and observed perplexity (see “Methods”). Fig 8 depicts the corresponding perplexity graph: With LDA hyper parameter optimization in place, an increasing number of topics allows to better predict the document collection. However, the gain lessens considerably beyond 50 topics. Therefore, we decided to work with n = 50 topics for further analysis.

Table 5 shows the inferred 50 topics, their marginal distribution, and the most relevant terms of the web pages (N = 3,746,055) of the top 3000 web sites (1000 for each ccTLD). The marginal distribution of a topic was measured by the probability that the topic was sampled from web pages, while the relevance of a term was measured by the probability that it was sampled from its topic. Word cloud representations of these topics can be found in S5 Appendix. The topics were summarized into 11 themes (see “Methods”). The most prevalent theme was related to “Research & Science”, followed by “Illness & Injury”, “The State”, “Healthcare structures”, “Diet & Food”, “Medical Specialities”, “Economy”, “Food production”, “Health communication”, “Family”, and “Other”.

**Table 5:** The 50 topics that were identified from the web pages of the top 1000 web sites for each ccTLD. The sample terms were ordered based on their relevance to the topic.

Theme	ID and Label	Most relevant terms	Distribution (probability)
**Research & Science**			
	T6 Clinical Trial	patient, study, treatment, diseas, clinical, research, abstract, effect, method, result, therapy, betwe, factor, health, journal, review, analysis, group, using, human, trial, during, level, disord, outcom, project, control, associated, chang, medical, model, medicin, protein, studi, tumor, mechanism, development, respons, surgery, expression, impact, molecular, infection, european, association, mutation, brain, function, different, chronic	0.0261
	T8 Funding	thema, klinik, untersuch, scientist-programm, promotionsstipendi, betreu, zusammenfass, untersucht, klinisch, patient, projekt, clinician, institut, studi, medizin, forder, infektion, moglich, inhalt, verschied, einfluss, funktion, wissenschaft, entwickl, human, bedeut, forschung, arbeit, laufzeit, mechanism, funktionell, advanced, erkrank, modell, medical, effekt, konnt, stell, therapi, wirkung, organag, bereit, spezif, method, ausschreib, antragstell, sepsis, vorlieg, regulation, gesund	0.0244
	T9 Efficacy Study	patient, studi, klinisch, behandl, haufig, erhoht, therapi, wirkung, wirksam, risiko, gleichzeit, erkrank, signifikant, monat, behandelt, taglich, schwer, beobachtet, empfohl, zeigt, dosis, effekt, kombination, unerwunscht, vergleich, verabreich, anwend, moglich, bekannt, gelegent, symptom, erhoh, untersucht, placebo, verabreicht, auftret, durchgefuhrt, berichtet, gering, unterschied, hinweis, fuhrt, zusatz, stund, ergebniss, system, kardiovaskular, diabet, niedrig, erwachs	0.0209
	T13 Human Biology & Genetics	forsch, wissenschaftl, konnt, studi, mensch, universitat, gehirn, genet, protein, natur, gross, menschlich, pflanz, untersucht, ergebniss, bestimmt, entdeckt, unterschied, bereit, verschied, erklart, zeigt, untersuch, wichtig, biolog, bekannt, bakteri, klein, sogenannt, entwickelt, molekular, verandert, grupp, ahnlich, university, erstmal, method, molekul, entsteh, wissenschaft, genau, nervenzell, enzym, einzeln, forschung, entdeck, entwickeln, million, kolleg, genom	0.0221
	T29 Science Communication	pressemitteil, entwickl, unternehm, entwickelt, technologi, bereich, system, digital, anwend, innovativ, bietet, einsatz, produkt, information, technisch, losung, medizintechn, weltweit, industri, herstell, verfahr, schnell, softwar, innovation, moglich, forschung, fraunhof, fraunhofer-gesellschaft, ermoglicht, stellt, sensor, markt, elektron, technik, wirtschaft, integriert, gerat, fraunhofer-institut, robot, zukunft, modern, anforder, fuhrend, erfolgreich, wissenschaft, mobil, deutschland, aktuell, medizin, comput	0.0215
	T33 Medical Newspaper	medizin, aktuell, patient, deutsch, studi, kommentar, anzeig, rubrik, polit, artikel, newslett, archiv, wissenschaft, perspektiv, thoraxchirurgi, servic, versorg, autor, berlin, anmeld, arztestell, nachricht, cannabis, gesundheitspolit, diabet, fachgebiet, operi, english, registriert, krankenhaus, edition, arzteschaft, praxis, ernahr, deutschland, management, entwickelt, einsatz, merklist, registri, bildergaleri, behandl, videos, medizinreport, email, da-titel, thema, urologi, e-mail, onkologi	0.0140
	T37 Space Research	berlin, gross, genau, proband, experiment, beweg, forsch, zeigt, wissenschaftl, konnt, physik, raumfahrt, tandemx, klein, gebiet, moglich, schnell, unterschied, untersuch, aufnahm, geschwind, beobacht, struktur, messung, digital, terrasarx, richtung, objekt, oberflach, satellit, verander, wissenschaft, dynam, beispiel, weltweit, mission, findet, stark, sogenannt, schwarz, stern, sekund, elektron, abstand, planet, arbeit, kilomet, gleich, direkt, natur	0.0171
	T49 Medical Journals	ausgab, erhalt, zugang, zeitschrift, volltext, autor, journal, spring, articl, zusammenfass, verlag, original, originalpap, access, wiesbad, kapitel, erschi, chapt, wichtig, author, report, buchkapitel, fachmedi, full-text, published, heidelberg, hinweis, publish, conferenc, sonderheft, mitteil, abstract, leitthema, editorial, berlin, inhaltsverzeichnis, editor, fortschritt, version, review, fortbild, michael, beitrag, pdf-version, thomas, originali, current, herunterlad, david, without	0.0150
**Illness & Injury**			
	T5 Oncology	tumor, thoraxchirurgi, klinik, patient, forschung, gutart, kontakt, notfall, sitemap, herzchirurgi, ursach, entsteh, bosart, therapi, haufig, pleuraerguss, brustwand, willkomm, lungenkreb, entfern, kardiotechn, pleuraempy, geweb, lungenmetastas, ambulanz, diagnost, lungentumor, metastas, symptom, moglich, brustwandtumor, hyperhidrosis, entfernt, beruf, operativ, angehor, operation, karri, erkrank, studium, uniklinikum, zuweis, brustfelltumor, hauptmenu, mediastinaltumor, pneumothorax, gross, untersuch, institut, mukoviszidos	0.0180
	T19 Accidents & Injuries	jahrig, mensch, polizei, schwer, gefahr, verletzt, berlin, erklart, bereit, stadt, unfall, berichtet, nachricht, freitag, mittwoch, dienstag, donnerstag, montag, betroff, krankenhaus, angab, vergang, kommentar, anzeig, sonntag, fluchtling, video, einsatz, deutschland, gross, schul, musst, teilt, verdacht, samstag, monat, feuerwehr, zeigt, anseh, bekannt, sieht, sofort, stund, behord, sprech, todlich, ermittl, warnt, strass, focus	0.0197
	T21 Chronic Illness	symptom, infektion, erkrank, haufig, krankheit, betroff, allergi, entzund, therapi, chronisch, ursach, bakteri, mensch, antibiotika, allerg, gesund, erreg, behandl, medikament, diagnos, auslos, beschwerd, gefahr, immunsyst, asthma, medizin, kommt, moglich, ernahr, morbus, vorbeug, schwer, auftret, durchfall, beispiel, schutz, verlauf, neurodermitis, erkalt, schmerz, ubertrag, typisch, hepatitis, vitamin, diabet, tipps, bakteriell, juckreiz, information, deutschland	0.0159
	T26 Pain	schmerz, beweg, behandl, therapi, gelenk, moglich, ursach, acumax, ubung, verletz, inhalt, betroff, beschwerd, haufig, patient, medizin, knoch, kursleit, verschied, bereich, ruckenschmerz, erkrank, muskeln, stark, arthros, wirbelsaul, operation, kommt, chronisch, anatomi, belast, akupunktur, information, therapeut, massag, technik, speziell, training, method, sport, schult, druck, unterstutz, unterschied, muskulatur, orthopad, symptom, geweb, stell, arbeit	0.0281
	T28 Heart diseases	herzinsuffizienz, entwickl, hypertrophi, einfluss, verander, mitochondrial, forschung, vermindert, insulinresistenz, untersuch, patient, insulinempfind, kardial, projekt, thoraxchirurgi, verschied, bekannt, sirtuin, chronisch, biogenes, reduziert, pgc-1alpha, expression, untersucht, erhoht, sitemap, druckuberlast, vermehrt, genet, vermut, studi, ausbild, notfall, klinik, intrins, forschungsgebiet, niedrig, arbeitslast, fettsaureoxidation, glukoseoxidation, mitochondri, moglich, verbund, fuhrt, kommt, auftret, klinisch, entsteh, diabet, beeinflusst	0.0171
	T31 Mental Illness	psychisch, storung, depression, psychotherapi, psychiatri, verweist, jugend, psychosomat, therapi, behandl, betroff, angst, therapeut, mensch, patient, person, erwachs, erkrank, psychotherapeut, psycholog, psychiatr, demenz, medizin, belast, verhalt, verhaltensstor, lexikon, depressiv, haufig, berat, angehor, krankheit, sozial, ursach, problem, gesund, schwer, seelisch, symptom, urban, schizophreni, krank, psychologi, emotional, psychiat, karnt, diagnos, diagnost, psychosozial, essstor	0.0179
	T35 Pandemic & Vaccination	impfung, virus, studi, impfstoff, coronavirus, todesfall, mensch, infiziert, deutschland, schweinegripp, gesund, schweiz, osterreich, schutz, infektion, erkrankt, expert, gripp, gefahr, deutsch, ebola, geimpft, krankheit, china, aktuell, impfpflicht, ansteck, hpv-impfung, macht, wirksam, million, robert, moglich, erreg, autismus, zusammenhang, europa, ubertrag, weltweit, ausbruch, impfkrit, ungeimpft, gemeldet, nebenwirk, berlin, krank, stiko, hepatitis, schwer, covid	0.0122
	T42 Respiratory & pulmonary diseases	erkrank, patient, chronisch, rauch, ursach, symptom, klinik, forschung, thoraxchirurgi, kommt, kontakt, atemweg, belast, weiterhin, mukoviszidos, notfall, therapi, organ, sitemap, atemnot, betroff, beruf, herzchirurgi, auswurf, schwer, schleim, monat, sogenannt, vorhand, stark, geweb, entsteh, diagnost, lungenerkrank, angehor, willkomm, entzund, fuhrt, statist, ambulanz, emphys, moglich, verlauf, kardiotechn, wichtig, lungenkreb, obstruktiv, lungenemphys, genannt, erkrankt	0.0144
	T46 Neurological diseases	projekt, multipl, gehirn, e-mail, kontakt, betreu, klinik, neurologi, thiem, motor, patient, verander, kognitiv, erkrank, skleros, alter, parkinson, untersuch, projektbeschreib, method, mechanism, funktionell, organ, neuron, verschied, einfluss, information, mikroglia, adult, stammzell, untersucht, studi, fahig, stress, analys, moglich, infection, process, neurogenes, aktivitat, prozess, beeintracht, neurolog, neurodegenerativ, altersbedingt, bereit, funktion, regeneration, altersabhang, diseas	0.0138
**The State**			
	T1 Healthcare system	anzahl, anteil, krankenhaus, versorg, behandl, klinik, direkt, beschaftigungsverhaltnis, untersuch, fachabteil, krankheit, operation, ambulant, vollkraft, diagnost, stationar, ambulanz, medizin, patient, sonstig, therapi, durchschnitt, krankenhaussuch, betreu, weiterempfehl, fallzahl, stund, pflegekraft, verfugbar, pfleg, vollstationar, arztsuch, startseit, krankenhausaufenthalt, arztlich, information, chirurgi, arztinn, erlauter, verletz, zufried, haufig, pfleger, ergebnis, intensivmedizin, person, erganzt, angestellt, verhaltnis, massgeb	0.0797
	T4 Sociology & society	mensch, gesellschaft, sozial, person, macht, natur, beispiel, sprach, bezieh, begriff, verhalt, arbeit, gefuhl, gross, erfahr, moglich, wissenschaft, polit, theori, wirklich, menschlich, situation, sexuell, bewusst, famili, bestimmt, bedeut, jahrhundert, unterschied, gewalt, spiel, einfach, vorstell, beobacht, sprech, lasst, versteh, wahrnehm, erklar, ausdruck, gedank, angst, kommt, heisst, recht, steht, freud, einmal, verschied, geschlecht	0.0404
	T7 Law	entscheid, gesetz, recht, urteil, gericht, regel, grundsatz, bverfg, person, verfahr, offent, grund, beschwerdefuhr, moglich, gesetzgeb, bestimm, richt, zulass, vorlieg, erwag, interess, anspruch, rechtlich, beschwerd, tatsach, vorschrift, allgemein, bundesgericht, bereit, massnahm, zustand, voraussetz, geltend, verfug, antrag, umstand, betroff, schutz, besond, erheb, kanton, besteh, entsprech, begrund, partei, deutsch, einzeln, bestimmt, beschluss, ausdruck	0.0299
	T18 Legal affairs	absatz, inhaltsverzeichnis, zustand, artikel, anlag, verordn, prufung, nichtamt, person, fassung, zulass, angab, erford, behord, europa, gesetz, anforder, genannt, richtlini, anhang, anwend, abschnitt, massnahm, vorschrift, ander, durchfuhr, folgend, betrieb, buchstab, della, stell, verfahr, verbind, sonstig, bestimm, stoff, verwend, information, genehm, bestimmt, zweck, verkehr, unternehm, rechtsverordn, herstell, zugelass, parti, nachweis, tatig, antrag	0.0222
**Healthcare Structures**			
	T15 Health insurance	leistung, gesetz, krankenkass, arbeitgeb, anspruch, versichert, regel, person, beitrag, betrieb, tatig, arbeit, schul, arbeitnehm, monat, beschaftigt, mitglied, versorg, pfleg, beruf, absatz, gemeinsam, voraussetz, erhalt, antrag, massnahm, moglich, vereinbar, einricht, besond, zustand, aufgab, grund, krankenversicher, grundsatz, gesellschaft, erford, unternehm, arztlich, stell, ander, verein, berat, besteht, privat, entsprech, berucksicht, personal, allgemein, zusatz	0.0205
	T23 Medical Education	universitat, wissenschaft, deutsch, forschung, medizin, institut, international, gesellschaft, ausbild, munch, fakultat, gotting, studier, arbeit, hochschul, professor, berlin, bereich, gemeinsam, projekt, vortrag, studium, email, deutschland, student, zusammenarbeit, veranstalt, beruf, seminar, forder, entwickl, mitglied, bildung, magdeburg, programm, grundlag, teilnehm, hamburg, national, kooperation, information, dissertation, unterstutz, tatig, munst, abteil, bewerb, chemi, erfolgreich, stiftung	0.0198
	T24 Research at University hospitals	forschung, klinik, medizin, zentrum, klinisch, mitarbeit, patient, aktuell, information, pfleg, kontakt, studi, publikation, institut, heidelberg, schwerpunkt, fortbild, veranstalt, ambulanz, station, frankfurt, diagnost, sprechstund, allgemein, stationar, interdisziplinar, universitatsklinikum, zentral, therapi, weiterbild, aufenthalt, leistung, geburtshilf, labor, neurochirurgi, anfahrt, uberblick, ambulant, onkologi, molekular, immunologi, zuweis, aufnahm, neurologi, radiologi, abteil, leistungsspektrum, qualitatsmanagement, spend, willkomm	0.0202
	T25 Institutes at University hospitals	klinik, therapi, diagnost, poliklin, medizin, martin, koradi, institut, universitatsklinikum, erkrank, palliativ, ambulanz, abteil, phytotherapi, weiterbild, krankenpfleg, angebor, pflegeheim, onkologi, department, psychiatr, spitex, universitatsklin, spital, leistung, behandl, hamatologi, jugend, zentrum, padiatri, jugendmedizin, krankheit, dermatologi, padiatr, nuklearmedizin, strahlentherapi, klinisch, rheumatologi, schwerpunkt, allergologi, endokrinologi, untersuch, gastroenterologi, patient, universitatsmedizin, krankenhaus, interdisziplinar, immunologi, radiologi, pneumologi	0.0137
	T30 Online Pharmacy	arzneimittel, preis, behandl, angebot, preisvergleich, anwend, medikament, merkzettel, einnahm, praparat, apothek, information, produkt, paypal, stern, dosis, kategori, rechnung, versand, zahlungsart, erhalt, stuck, schwangerschaft, patient, preisalarm, tablett, einnehm, pharma, angewendet, informi, eingenomm, e-mail, gunstig, schwang, kreditkart, bewert, lastschrift, hilfstoff, infos, auftret, grundpreis, herstell, vorkass, apomio, risiko, filmtablett, deutschland, arzneimitteln, einmal, tiermedizin	0.0216
	T32 (Online) Appointment & Telemedicine	erkrank, extern, einricht, telefon, zentrum, linkwebseit, sprechzeit, genet, vereinbar, mensch, druck, krankenhaus, deutsch, universitatsklinikum, behandelt, se-atlas, bietet, benutzerprof, berat, bronchialkarzinom, diagnost, patient, kartenmarkier, therapi, klinik, pfeil, national, eintrag, kontakt, korrekt, massnahm, gefordert, klinisch, referenznetzwerk, javascript, bedingt, berlin, diagnos, aktivi, aktionsplan, darzustell, klinikum, sprechstund, versorgungsangebot, bundestag, beschluss, eingeb, adress, umschlag, folgend	0.0142
	T36 Hospital clinics	klinik, campus, medizin, lubeckerwachs, intensivmedizin, lubeckkind, kardiologi, angiologi, sprechstund, jugendlicheerwachs, orthopadi, geburtshilf, gynakologi, ambulanz, jugend, frauenheilkund, unfallchirurgi, institut, lubeck, therapi, gynakolog, jugendmedizin, ergebniss, labor, ereigniss, notfall, brustzentrum, anasthesiologi, kompetenzzentr, chirurgi, schmerzambulanz, onkologi, vergleich, statist, erwartet, fachlich, bundesweit, trend, untersucht, interpretation, herzschrittmach, referenzbereich, gezahlt, entwickl, vorjahr, qualitatsindikator, sportmedizin, rechner, risikoadjustiert, iqtig	0.0155
	T50 University Hospital clinics	campus, klinik, kielerwachs, intensivmedizin, anasthesiologi, brustzentrum, medizin, operativ, institut, ambulanz, sprechstund, therapi, ergebniss, labor, privatsprechstund, patient, schmerztherapi, notfall, jugendlicheerwachs, radiologi, aufnahm, onkologi, kompetenzzentr, schmerzambulanz, steinfath, besuch, praoperativ, hamatologi, neuroradiologi, herzfehl, gesund, vacuumbiopsiesprechstund, schwerpunkt, tumorrisikosprechstund, zahnarzt, brusterkrank, lubeckkind, angebor, station, aufklarungssprechstund, brust-rekonstruktionssprechstund, mensch, prothet, leukami, information, kinderkardiologi, psychologi, nachsorg, kielkind, soziologi	0.0078
**Diet & Food**			
	T2 Food intolerance	vitamin, symptom, medizin, aufnahm, kommt, klingt, eiweiss, kohlenhydrat, vitamin-b12-mangel, sorbit, sorbitunvertrag, chirurgi, orthopad, allgemeinmedizin, gesund, behandl, information, krankheit, wahlarzt, mangel, hilft, oftmal, rechtzeit, erlaubt, erkenn, fallt, nimmt, ahnlich, erfullt, einzig, ausgesproch, geschieht, unverzichtbar, wiederum, lebenswicht, beheb, einschrank, unangenehm, beinah, glutamat, aufzunehm, mittel, konsum, unglaub, verdau, blahung, wurst, abnehm, erzeugt, erschw	0.0415
	T12 Healthy lifestyle & nutritional counseling	gesund, ernahr, mensch, diabet, studi, sport, ubergewicht, schlaf, alkohol, richtig, schnell, macht, trink, enthalt, taglich, regelmass, beweg, prozent, wichtig, stress, tipps, haufig, erhoht, gefahr, hilft, risiko, abnehm, rauch, natur, einfach, lebensmittel, wirkung, stark, schlecht, kommt, nahrung, gewicht, expert, positiv, training, gesundheit, verzicht, gross, beispiel, kaffe, thema, vitamin, ausreich, stund, lasst	0.0199
	T39 Food ingredients	fettsaur, sonst, vitamin, einheit, inhaltsstoff, aminosaur, essent, eiweiss, lebensmittel, ballaststoff, mineralstoff, einfach, folsaur, kohlenhydrat, polysaccharid, langkett, kurzkett, brotein, monosaccharid, mittelkett, oligosaccharid, disaccharid, zuckeralkohol, resorb, allerg, zusatzstoff, magnesium, calcium, pflanzlich, gramm, kalium, tierisch, zuruck, natrium, bestandteil, spurenelement, freie, entsprech, gesamt, mehrfach, glucos, speziell, cholesterin, stark, arginin, organ, aktiv, gesattigt, verzehr, alkohol	0.0125
	T41 Cooking recipes	bewert, normal, simpel, schneid, klein, rezept, minut, pflanz, gemus, frisch, wasch, einfach, schal, blatt, fleisch, gross, schnell, frucht, zutat, pfeff, tomat, milch, stuck, pfann, geschmack, abgegeb, knoblauch, kartoffeln, zwiebel, kraut, heiss, zubereit, scheib, zwiebeln, gefullt, honig, verwend, salat, enthalt, vegetar, anschliess, apfel, trock, anbrat, erhitz, vegan, olivenol, gewurz, verwendet, wurfeln	0.0097
**Medical Specialities**			
	T20 Therapies in Cardiology	patient, studi, klapp, klinik, thoraxchirurgi, eingriff, durchgefuhrt, herzchirurgi, erkrank, herzklapp, mechan, gross, operation, torst, biolog, herzchirurg, moglich, vorteil, aortenklapp, deutschland, risiko, haufig, thuring, gering, chirurg, universitatsklinikum, behandl, bypass, untersucht, notfall, kardiotechn, medizin, ergebniss, sitemap, forschung, krankenhaus, fuhrt, direktor, gemeinsam, wichtig, ersatz, reparatur, koronar, jahrig, material, erhoht, heinrich, klein, generation, schnell	0.0116
	T48 Treatment planning in Cardiology	patient, behandl, prozent, aktuell, wochenubersicht, haufig, herzinsuffizienz, termin, servic, pharmazi, herzchirurgi, modern, leitlini, torst, konventionell, medizin, verfahr, klinisch, arzneistoff, pz-markt, erkrank, bereit, genannt, newslett, wirtschaft, entwickl, stand, herzchirurg, pta-forum, studi, bereich, therapieoption, koronar, verschied, empfehl, infos, produkt, impressum, therapi, operativ, derzeit, minimal-invasiv, herausforder, rss-feed, folgend, erfolgt, figulla, herzerkrank, endokarditis, mediadat	0.0101
	T10 Medication information	medikament, arzneimittel, nebenwirk, anwend, arztin, wirkstoff, behandl, wirkung, einnahm, apothek, tablett, enthalt, dosier, angewendet, erhalt, produkt, auftret, eingenomm, einnehm, filmtablett, erfahr, schwangerschaft, stund, beacht, dosis, informi, folgend, wechselwirk, erhoht, preis, apothekerin, vorsicht, packung, taglich, patient, schwer, mittel, stillzeit, gleichzeit, sandoz, allerg, reaktion, haufig, warnhinweis, arztlich, erbrech, moglich, rezept, packungsgross, gegenanzeig	0.0327
	T34 Homeopathy	produkt, apothek, stund, pfleg, versandbereit, trock, gesund, homoopathi, vitamin, natur, erfahr, anwend, wirkung, arzneimittel, mittel, rezept, heilpflanz, ather, homoopath, kosmet, erkalt, warenkorb, korperpfleg, mlinnerhalb, preis, reinig, ernahr, gesicht, schussl, beruh, empfind, websit, versand, pflanzlich, pharmazi, enthalt, wirkt, diabet, allergi, gelenk, hautpfleg, kapseln, pharmazeut, krankheit, sonnenschutz, nagel, fitness, famili, inhaltsstoff, newslett	0.0157
	T38 Plastic surgery	profil, aufruf, chirurgi, asthet, facharzt, plastisch, function, dusseldorf, erfahr, behandl, zahnarzt, patient, preis, bericht, terminanfrag, eingriff, reaktion, werbung, implantat, chirurg, return, weiterleit, windowsettimeoutinit, operation, spezialist, windowpark, windowsettimeoutfunction, termin, zahnmedizin, praxis, medizin, jameda, munch, anzeig, freundlich, wartezeit, beispiel, durchgefuhrt, brustvergrosser, tweet, moglich, bochum, erfahrungsbericht, entfern, verfasst, thema, kompetent, zufried, fettabsaug, hamburg	0.0146
**Economy**			
	T3 Work & process organization	arbeit, sozial, unterschied, moglich, abbild, schul, unternehm, entwickl, verschied, method, bedeut, begriff, folgend, einzeln, grundlag, bereich, zusammenhang, ansatz, ergebniss, person, beispiel, analys, prozess, mitarbeit, aspekt, darstell, untersuch, konzept, modell, definition, kapitel, individuell, organisation, information, aufgab, vergleich, leseprob, faktor, wichtig, enthalt, stellt, grupp, management, gross, einfluss, auswirk, notwend, padagog, deutsch, entscheid	0.0316
	T11 Economic growth in Germany	prozent, deutschland, deutsch, europa, million, mensch, polit, berlin, gross, bereit, wirtschaft, international, vergang, derzeit, milliard, stark, bevolker, fordert, weltweit, insgesamt, offent, staat, kunftig, jahrlich, gesellschaft, osterreich, erklart, massnahm, unternehm, bundesregier, finanziell, betroff, studi, aktuell, markt, national, statist, betont, knapp, zeigt, global, befragt, heisst, unterstutz, sieht, zunehm, durchschnitt, grosst, konnt, regier	0.0239
	T17 Production of medical products	gross, energi, herstell, moglich, verwendet, stoff, chemisch, temperatur, klein, zusatz, enthalt, kommentar, elektr, verwend, strom, eigenschaft, material, beispiel, gerat, einsatz, eingesetzt, lasst, verbind, gering, natur, unterschied, flussig, verschied, oberflach, druck, einfach, materiali, besteht, geeignet, direkt, metall, hergestellt, speziell, anwend, licht, vorteil, anlag, schnell, reinig, benotigt, bereit, produkt, technisch, bearbeitet, kunststoff	0.0274
**Food production**			
	T14 Environmental protection in agriculture	projekt, landwirtschaft, flach, diffus, beitrag, notwend, gewass, schweiz, massnahm, untersuch, konzept, pestizid, oberflachenabfluss, gewasserbelast, agrochemikali, method, gebiet, arbeit, besteh, belast, zusammenarbeit, ressourc, untersucht, wirkung, raumlich, monitoring, folgend, vorschlag, forschungsprojekt, abschatz, dissertation, phosphor, projektbeginn, standortgerecht, ressourcenprojekt, okolog, erstellt, umwelt vergleich, schad, erheb, resultat, stamm, claudia, daniel, relevant, zuruck, unsich, zentral, vorgehc	0.0233
	T43 Pasture- and agriculture	erhol, verdichtet, umweltwirk, arbeitshoh, melkstand, untersucht, versuch, einfluss, agroscop, reduziert, analysiert, schult, schwein, regeneration, futter, bodenbearbeit, eingesetzt, einsatz, unterschied, positiv, zeigt, zweit, zusamm, verschied, erfasst, natur, entscheid, gering, angepasst, genau, wirkt, braucht, europa, bereich, zurich, jahrzehnt, auftrag, haufig, angelegt, verringert, beeinflusst, sekund, hundert, world, studi, kilogramm, pflanz, eingerichtet, optimiert, intensitat	0.0112
	T44 Food safety & consumer protection	ubersicht, lebensmittel, gesund, rechtsgrundlag, arbeitsschutz, produkt, untersuch, information, lebensmitteln, gentechn, tierisch, uberwach, arbeitsmedizin, fisch, tierhalt, zusatzstoff, bayer, kennzeichn, biolog, forschung, trinkwass, tiergesund, kontakt, uberpruf, projekt, getrank, lebensmittelsich, futtermittel, technisch, hinweis, bedarfsgegenstand, medizinprodukt, tierarzt, aufgab, mitglied, veranstalt, stoff, herkunft, chemisch, umwelt, akteur, pflanzlich, untersuchungsergebniss, umweltfaktor, allgemein, pravention, pflanzenschutzmittel, press, herstell, stellungnahmc	0.0108
	T47 Agricultural land use	landwirtschaft, zukunft, offenhalt, berggebiet, bedeut, berglandwirtschaft, verschied, auswirk, zeigt, entwickl, nutzung, flachennutz, wiesenbewasser, kulturlandschaft, flach, region, bericht, genutzt, betrieb, besteh, blick, ermoglicht, wissensluck, bergregion, schliess, kalbermast, leistung, biodiversitat, flachendeck, einkomm, nachhalt, kanton, multifunktional, deckungsbeitrag, regional, agrimontana, kulturflach, minimalnutzungsverfahr, minimalnutz, standort, intensiv, wichtig, zentral, schutz, stellt, gesamt, stand, gross, hintergrund, negativ	0.0093
**Health Communication**			
	T22 Health (disussion) forum	antwort, beitrag, thema, artikel, erfahr, kommentar, konnt, einfach, richtig, probl, medikament, krankheit, wirklich, weiss, bekomm, weiterles, hallo, forum, vielleicht, gesund, gruss, empfehl, gross, monat, einmal, stark, problem, schwer, zufried, twitt, stress, natur, moglich, schreib, angst, kommt, hilft, genau, eigent, stell, vorher, hilfreich, meinung, faktor, zitat, nebenwirk, schlecht, macht, gemacht, behandl	0.0156
	T27 Doctor rating portal	bewert, detail, praxis, kontakt, geschloss, e-mail, webseit, psychotherapi, offnet, partn, medizin, geoffnet, berat, montag, physiotherapi, zentrum, allgemeinmedizin, region, psycholog, heilprakt, therapi, schliesst, praktisch, strass, ganzheit, coaching, information, hypnos, genau, hamburg, psychotherapeut, heilpraktikerin, ergotherapi, nurnberg, homoopathi, orthopadi, gesund, osteopathi, system, wurzburg, innenstadt, behandl, facharzt, zahnarzt, naturheilkund, logopadi, berlin, munch, mensch, klassisch	0.0180
**Family**			
	T16 Pregnancy & family planning	schwangerschaft, untersuch, geburt, erkrank, haufig, moglich, ursach, risiko, schwang, betroff, operation, hormon, symptom, medikament, wichtig, brustkreb, krankheit, diagnos, brust, erhoht, kommt, gross, beschwerd, therapi, schlaganfall, behandl, blutung, medizin, klein, risikofaktor, gefass, gesund, auftret, komplikation, weiblich, herzinfarkt, normal, vorsorg, besteht, sogenannt, information, darmkreb, funktion, tumor, gebarmutt, verander, durchgefuhrt, schilddrus, chemotherapi, eingriff	0.0138
**Other**			
	T40 —^a^	ebook, klick, michael, thomas, empfehl, medizin, christian, kommentar, ubertrag, facebook, aktiv, twitt, andreas, verlag, aktivi, googl, preis, datenschutz, schreib, artikel, lokal, stefan, button, martin, christoph, wolfgang, johann, markus, frank, information, stuttgart, inhalt, klaus, auflag, deutsch, departement, georg, matthias, susann, verfass, barbara, daniel, ulrich, video, andrea, sabin, erfahr, publikationslink, alexand, bernhard	0.0104
	T45 —^a^	artikel, schweiz, zurich, spektrum, anzeig, thema, verlag, frank, lexikon, mail-black, whatsapp-black, print-black, twitter-black, facebook-black, mensch, gemeind, basel, verwandt, konnt, minut, druck, redaktion, spital, kanton, empfehl, aktuell, inhalt, lesedau, heidelberg, informi, beantwort, startseit, verstandnis, interessi, aargau, arrow-right, lesenswert, kommentar, thumb-upja, thumb-downnein, e-mail, biologi, akadem, anmerk, copyright, kompakt, hinterleg, bundesrat, amazond, lexika	0.0132

^a^ Could not be named by the volunteers.

The theme “Research & Science” covered eight topics: “Clinical Trials” (T6), “Funding” (T8), “Efficacy Studies” (T9), “Human Biology & Genetics” (T13), “Science Communication” (T29), “Medical Newspaper” (T33), “Space Research” (T37), and “Medical Journals” (T49).

“Illness & Injury” contained nine topics: “Oncology” (T5), “Accidents & Injuries” (T19), “Chronic Illness” (T21), “Pain” (T26), “Heart diseases” (T28), “Mental Illness” (T31), “Pandemic & Vaccination” (T35), “Respiratory & pulmonary diseases” (T42) and “Neurological diseases” (T46). Among them were topics related to the most common diseases in the D-A-CH region (T5, T21, T26, T31, T42, T46) [79]. It also contained one topic (T35) referring to the COVID-19 pandemic and one topic (T19) about (car) accidents and related injuries. The theme “Medical Specialities” covered topics related to “Therapies in Cardiology” (T20), “Treatment planning in Cardiology” (T48), “Medication information” (T10), “Homeopathy” (T34) and “Plastic surgery” (T38). In addition, a theme “Familiy” containing only one topic “Pregnancy & family planning” (T16) was found.

“The state” covered four topics about the “Healthcare system” (T1), “Sociology & society” (T4) and legal aspects related to healthcare (T7, T18). The closely related field “Healthcare structures” contained topics related to “Health insurance” (T15), “Medical education” (T23), “Research at University hospitals” (T24), “Institutes at University hospitals” (T25), “Online Pharmacy” (T30), “(Online) Appointment & Telemedicine” (T32), “Hospital clinics” (T36) and “University Hospital clinics” (T50).

The theme “Diet & Food” covered aspects such as “Food intolerance” (T2), “Healthy lifestyle & nutritional counseling” (T12), “Food ingredients” (T39) and “Cooking recipes” (T41). The closely related theme “Food production” covers topics such as “Environmental protection agriculture” (T14), “Pasture and agriculture” (T43), “Food safety & consumer protection” (T44) and “Agricultural land use” (T47). “Economy” covered topics “Work & process organization”, “Economic growth in Germany” (T17), and “Production of medical products” (T2).

In addition, we found a theme “Health communication” including two topics: “Health (disussion) forum” (T22), “Doctor rating portal” (T27). “Other” was assigned to T40 and T45, which could not be named by the volunteers.

**Fig 9:**
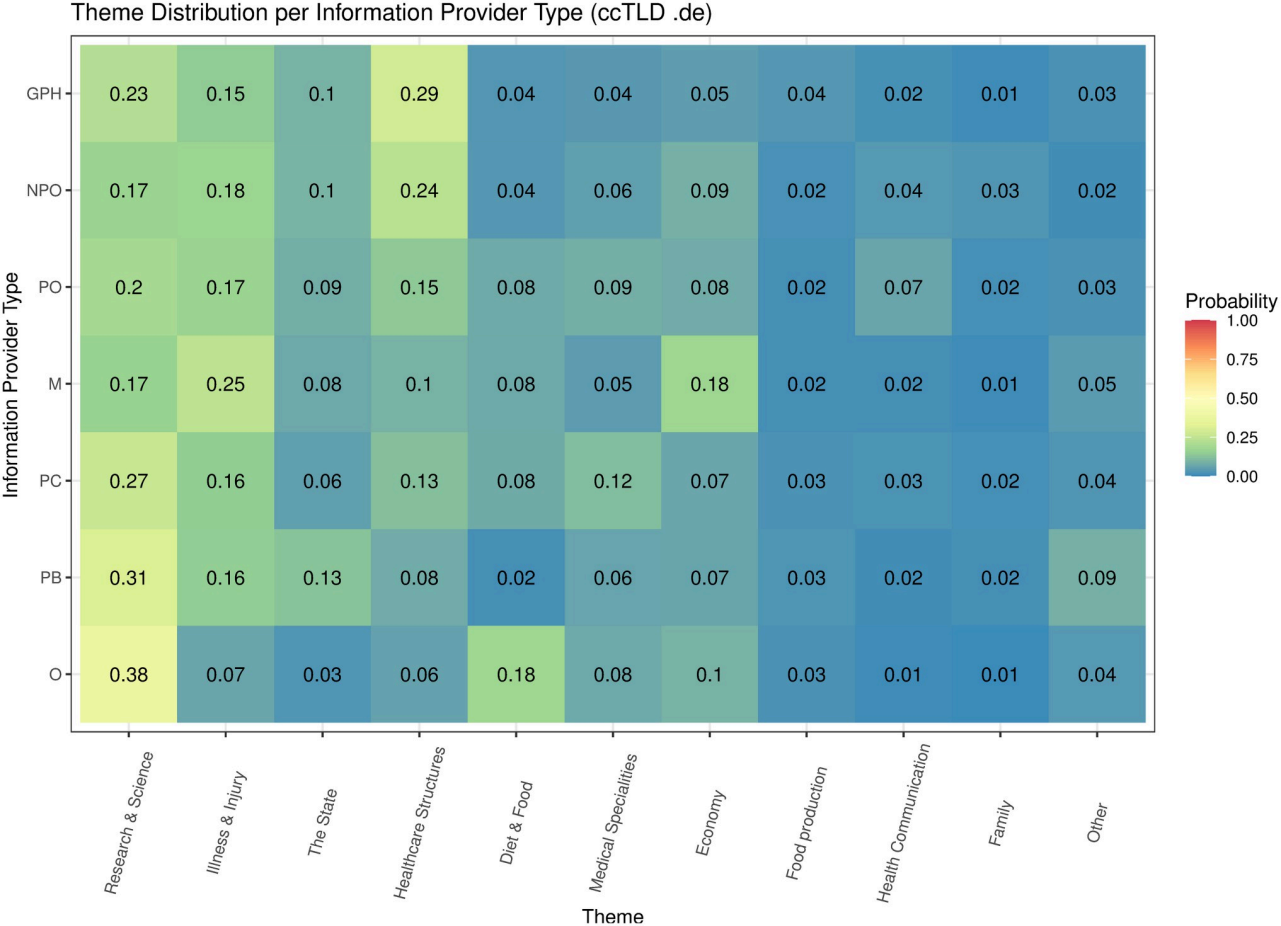
Theme distribution per information provider type for the ccTLD “.de”. Information provider types: GPH: Government, Public Institution or Public Health, NPO: Non-Profit Organization, PO: Private Organization, M: Mainstream or Local News, PC: Pharmaceutical Company, PB: Private Blog, Other: O.

**Fig 10:**
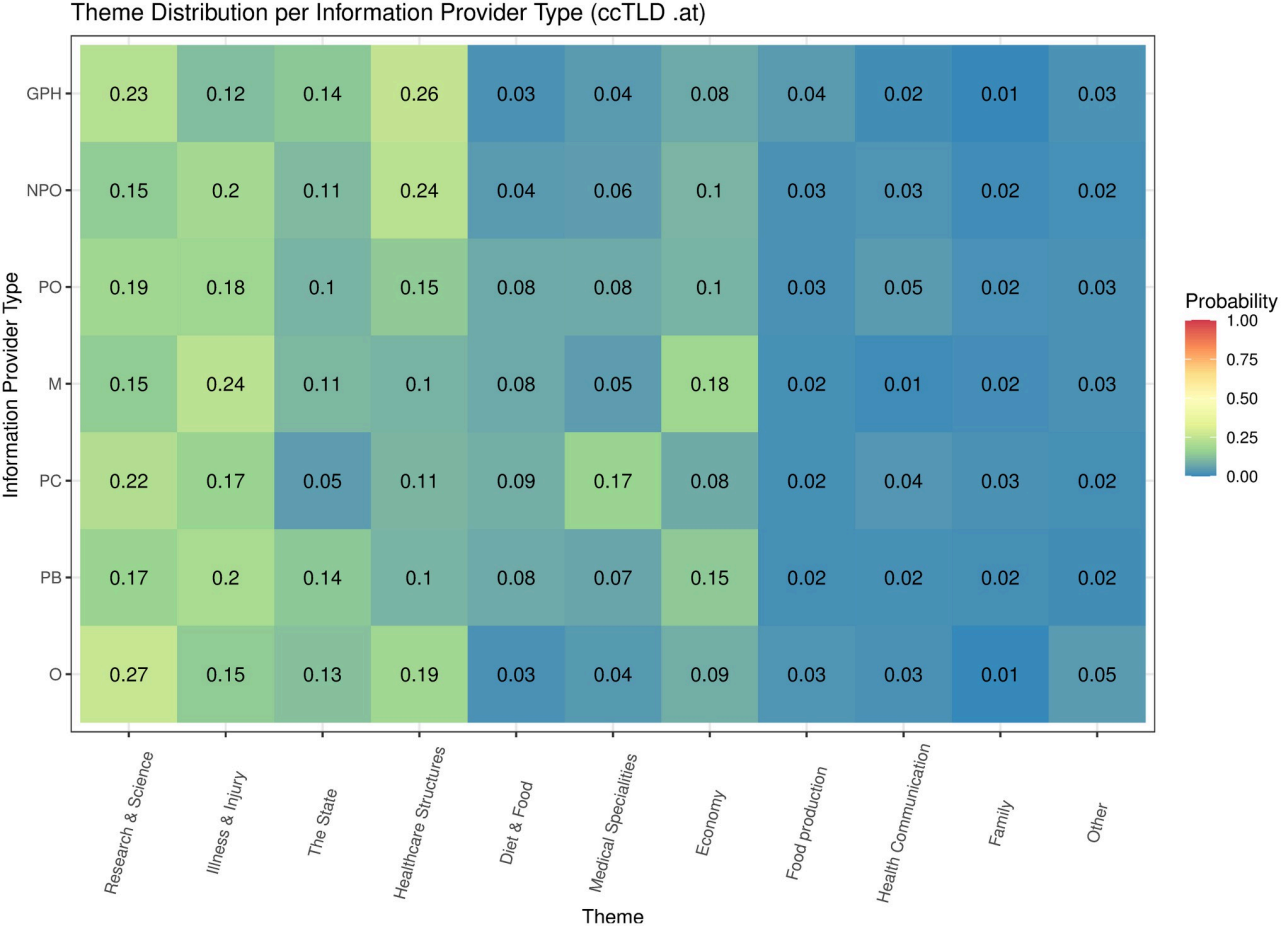
Theme distribution per information provider type for the ccTLD “.at”. Information provider types: GPH: Government, Public Institution or Public Health, NPO: Non-Profit Organization, PO: Private Organization, M: Mainstream or Local News, PC: Pharmaceutical Company, PB: Private Blog, Other: O.

**Fig 11:**
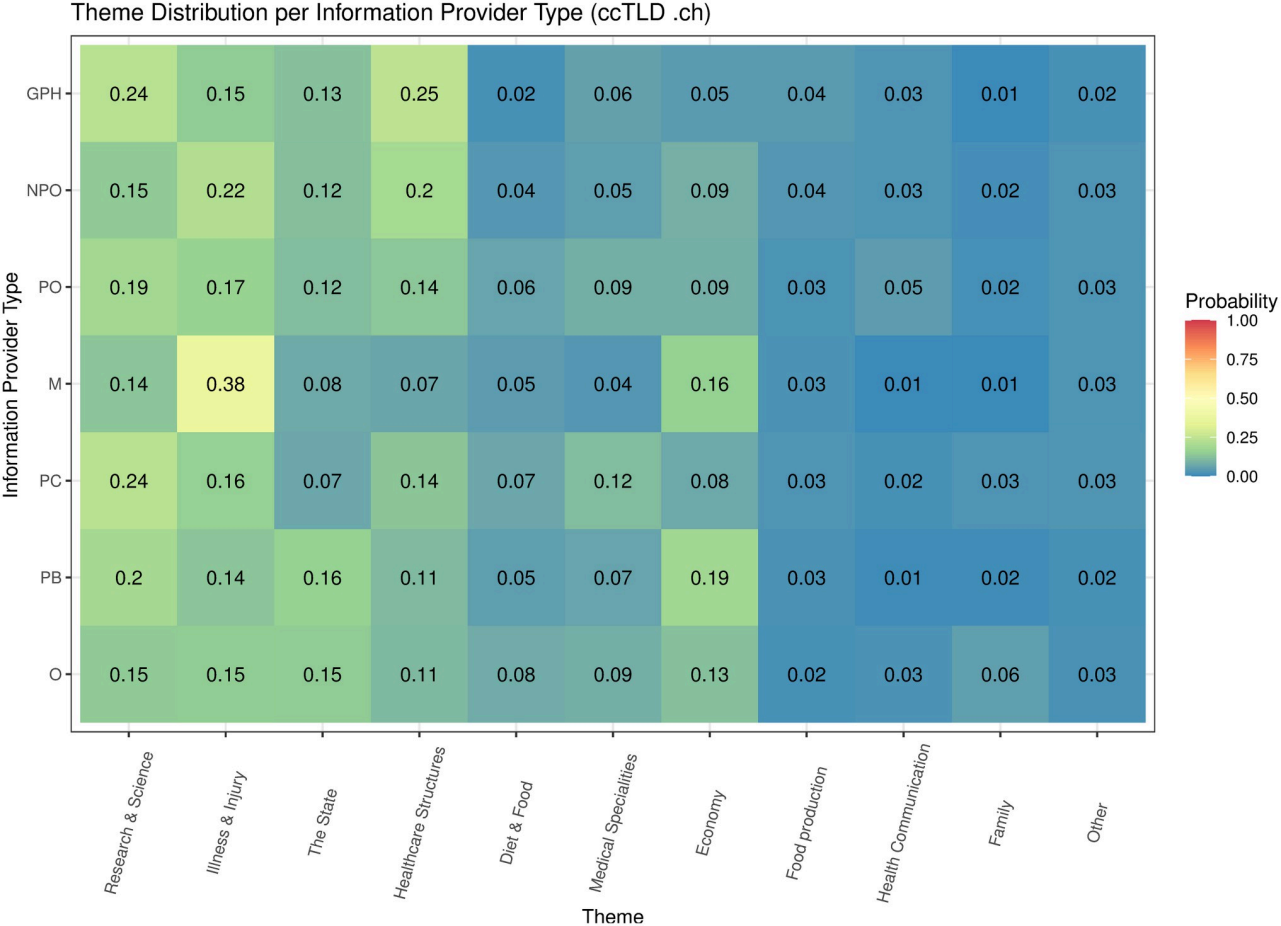
Theme distribution per information provider type for the ccTLD “.ch”. Information provider types: GPH: Government, Public Institution or Public Health, NPO: Non-Profit Organization, PO: Private Organization, M: Mainstream or Local News, PC: Pharmaceutical Company, PB: Private Blog, Other: O.

Figs 9–11 depict the theme distribution per information provider type for each ccTLD. The theme distribution for each ccTLD and for each information provider type seems to be similar between each country. Mainstream or local news agencies (M) report primarily on the topics "Illness and Injury" and "Economy”. Governmental or public (health) organizations (GPH), on the other hand, mainly focus on "Research & Science," "Healthcare Structures," and "Illness and Injury". In contrast, NPOs report predominantly on "Illness and Injury," followed by "Research & Science" and "Healthcare Structures". This is similar to the topic distribution for private organizations (POs) and pharmaceutical companies (PCs). Overall, it seems that the primary content of the sGHW across all ccTLDs is focused on "Research & Science," "Illness & Injury," and "Healthcare Structures".

**Fig 12:**
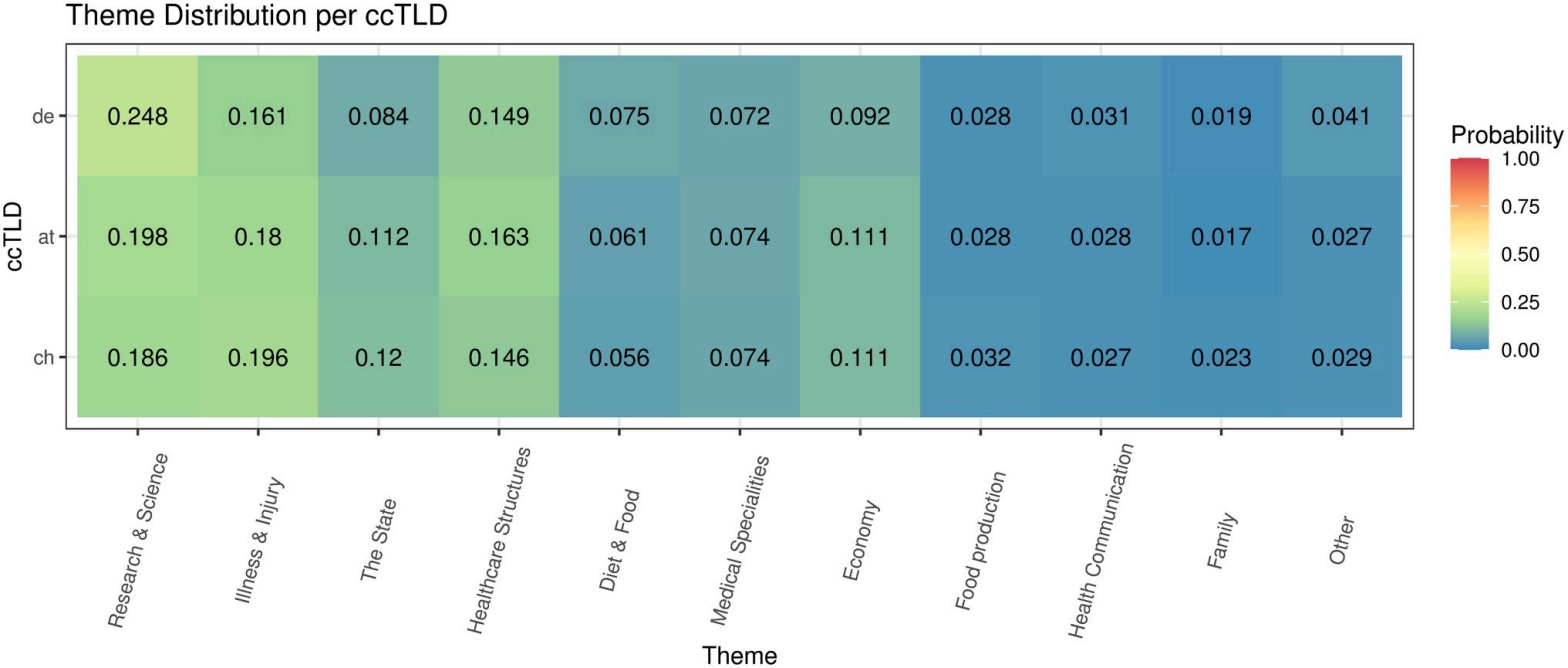
Theme distribution per ccTLD.

Fig 12 depicts the theme distribution per ccTLD. On average, the theme “Research & Science” accounts for a 21.04% (“Illness & Injury”: 17.92%; “Healthcare Structures”: 15.27%; “The State”: 10.52%; “Economy”: 10.50%; “Medical Specialities”: 7.30%; “Diet & Food”: 6.36%; “Other”: 3.35%; “Food production”: 2.94%; “Health Communication”: 2.90%; “Familiy”: 2.00%) of all topics across all ccTLDs and provider types. This suggests, that the content of the sGHW is similar between the countries of the D-A-CH region (at least for the ccTLDs studied) and that the information need of users may not vary greatly between the individual countries.

In addition, we computed the gini coefficient [67] for the topic distributions of each ccTLD: G(“.de”) = 0.763, G(“.at”) = 0.746, and G(“.ch”) = 0.748. These values indicate that the topics vary strongly between websites of a given ccTLD.

## Discussion

### Principal findings

The graph analysis (see study aim 1) shows that the sGHW is dominated by private stakeholders (54.03%; 1621/3000) followed by public institutions (18.50%; 555/3000) and nonprofit organizations (18.03%; 541/3000). However, looking at the top-ranked 25 web sites (see Tables 1–3), the majority of web sites originate from governmental or public (health) institutions (35%; 26/75) and non-profit organizations (16%; 12/75). “Mainstream or Local News” account for 15% (11/75). In addition, we were able to identify 50 abstract topics, that we summarized and grouped into 11 themes: “Research & Science”, “Ilness & Injury”, “The State”, “Healthcare structures”, “Diet & Food”, “Medical Specialities”, “Economy”, “Food production”, “Health communication”, “Family” and “Other”.

With respect to study aims 2 and 3, our readability analysis reveals that the majority of the collected web sites is difficult or very difficult (D+VD) to read (see S4 Appendix), as shown by the WSTF (84,63%; 2539/3000). This ratio is similar for each ccTLD: 86.20% (862/1000) for “.de”, 84.40% for “.at”, and 83.30% (833/1000) for “.ch”. This finding coincides with the outcome of the German adoption of the FRE scale: 2691/3000 (89.70%) web sites are D or VD. Again, the ratio is similar for each ccTLD: 88.30% (883/1000) for “.de”, 90.70% (907/1000) for “.at”, and 90.10% (901/1000) for “.ch”. Thus, health-related web sites are often written at high readability level and might not suit the intended group of readers. This is in line with the results of other studies, which also reported high readability levels for such resources [18–20, 22, 23, 26, 27].

Our vocabulary analysis revealed that 44.00% (1320/3000) web sites use vocabulary that is well suited for a lay audience. Again, the ratio is similar for each ccTLD: 48.50% (485/1000) for “.de”, 41.90% (419/1000) for “.at”, and 41.60% (416/1000) for “.ch”. This suggests that relatively few medical expert terms have been used on related web pages, or expert terminology has been actively avoided.

The distribution of in- and out-degrees i.e. links per host by rank is in line with the results from Meusel [48]. Although the latter publication analysed a large but unfocused crawl, the nature of its respective distribution is similar to ours. This suggests that the distribution of incoming and outgoing links in the sGHW is not different from the rest of the web.

We found that the sentence complexity measures FRE and WSTF are strongly correlated on health-related web pages such that they can be used interchangeably. Also, high vocabulary difficulty moderately correlates with sentence complexity. On average, the theme “Research & Science” accounts for a 21.04%; “Illness & Injury”: 17.92%; “Healthcare Structures”: 15.27%; “The State”: 10.52%; “Economy”: 10.50%; “Medical Specialities”: 7.30%; “Diet & Food”: 6.36%; “Other”: 3.35%; “Food production”: 2.94%; “Health Communication”: 2.90%; “Familiy”: 2.00% of all topics across all ccTLDs and provider types. This suggests, that the content of the sGHW is similar between the countries of the D-A-CH region (at least for the ccTLDs studied).

Overall, we demonstrated that a focused crawling approach and subsequent graph analysis can be leveraged to conduct a full scale readability and vocabulary assessment on a large sample of a language-specific part of the health-related web (study aim 4).

### Limitations

Several limitations apply for this study. First, we only considered the ccTLDs “.de”, “.at”, and “.ch” to avoid the need for a language classification system, as most web sites on these ccTLDs are written in German. Therefore, our dataset covers only a certain fraction of the GHW. For example (German) web sites published under “.com”, e.g. the web site of the electronic health record provider “www.vivy.com”, are not contained. In addition, our web crawl represents only a snapshot of the time when it was taken, i.e. web sites, which were created after the end of our crawl, are also not included in our dataset as we abstained from performing update operations to reduce computational complexity. A famous example for such a web site is the national health portal of Germany “gesund.bund.de” operated by the German Ministry of Health and released on 1^st^ September 2020.

Second, with a mean accuracy of 0.951 our classifier might have produced false positive results during the crawling process (see [13]). Third, we used a focused web crawling system to collect health-related web pages and to extract the raw text material from HTML content. For this reason, disturbance artifacts, such as different kinds of hyphens, XML fragments or misencoded characters, may still have been included in the extracted text material and thus have influenced our readability analysis. In addition, some analyzed web sites may only contain a small amount of (content) web pages which might lead to an either underestimated or overestimated average readability and/or vocabulary score (see S3 Appendix). This is due to the automatic nature of our web crawling process: (1) we omit (content) web pages, which were classified as non-relevant, (2) we respect crawler ethics (i.e., robots.txt), and (3) we are using an estimated priority value to determine crawling priority. Consequently, we might have missed additional relevant (content) web pages for a given website.

Next, we relied on the PageRank algorithm to determine a ranking of the most important web sites contained within the generated host-aggregated sGHW graph. This does not necessarily comply with the perspective of an individual user who is using a (commercial) search engine to find relevant health content nor does it correlate with visibility indices or “organic ranks” provided by (commercial) third party services. However, we think that ranking web sites based on PageRank, which was computed on the host-aggegated sGHW graph is justified as it is not biased by commercial interest and can be reproduced easily. Even more importantly, it is a well accepted approach to assess the importance of a graph node in graph theory [50, 82].

Moreover, detecting syllables is not a trivial task for the German language and is not always reliably [83]. As the adapted FRE and the WSTF are computed on the basis of the mean number of syllables per word, they can be influenced by the aforementioned inaccuracies. However, this applies to all NLP analysis tools for German text material. In addition, there is a lack of proper validation studies on the application of readability measures for German health-related text material. However, due to the frequent use of these instruments in the scientific community and their use by the German Agency for Quality in Medicine to assess the readability of their patient education guidelines and S3 guidelines [84], we consider them as a reference that allows comparisons of analyses of readability of health-related text material written in German.

Furthermore, solely computing the readability of text material disregards the individual knowledge and motivation of readers [63]. Aspects related to illustration and design were not included in the analysis. Consequently, the suitability of health-related web sites cannot exclusively be judged based on its readability or its used vocabulary [63]. Other methods, such as the Suitability Assessment of Materials (SAM) instrument [85] or DISCERN [86] go beyond measures of word and sentence length and cover other aspects of a web page that influence the understandability (or quality) of health information and text comprehension. However, these instruments require manual work and a sufficient number of judges to ensure an objective assessment. Moreover, with regard to our study, assessing 3,746,055 texts (i.e. web pages) would impose very high financial and human resources, which is not feasible.

### Comparison with prior work

#### Readability of health information material

Previous studies investigated the readability of health-related web pages [18, 26, 27] or the vocabulary difficulty of health education material provided as PDF brochures [24, 25].

In contrast to McInnes and Haglund [26] or Worrall et al. [27], we obtained our data collection by using a specifically trained focused web crawler [13] instead of retrieving it via a (commercial) search engine provider such as Google. Thus, our data collection is not influenced by commercial interests.

McInnes and Haglund [26] analyzed 352 web sites and computed a mean FRE of 46.08, which is difficult to read. In 2020, Worrall et al. [27] report that “only 17.2% [(n = 165)] of web pages [related to COVID-19 were written] at a universally readable level.” These findings are supported by Brütting et al. [18] who found low readability scores for 45 prominent web sites on melanoma immunotherapy written in German. These results are in line with our findings which reveal that the majority of the collected web sites is difficult or very difficult (D+VD) to read (see S4 Appendix).

In a previous study [61], Keinki et al. analyzed information booklets for German cancer patients. The authors found a mean vocabulary score of L = 5.09 signaling a higher difficulty for lay people. Wiesner et al. [25] found a mean vocabulary score of L = 3.66 for health education materials on Psoriasis/Psoriatic Arthritis written in German, indicating the use of less complex medical terminology. In contrast to the aforementioned studies, our study revealed higher mean vocabulary scores: L = 6.340 (SD = 2.572) for “.de”, L = 5.796 (SD = 2.543) for “.at”, and L = 5.885 (SD = 2.499) for “.ch”. This difference might result from the fact that we focused on health-related material contained in the GWH rather than limiting our study to patient information material only. Consequently, our data collection might contain web pages targeting (medical) experts, who make use of (medical) expert vocabulary.

#### Topic modeling on health information material

Previous studies applied topic modeling techniques to a variety of health information material such as content posted on social media, online newspaper articles or on web sites in general [36–42]. Most of these studies [38–42] focused on a specific health-related topic such as “hearing loss“, “weight loss“, “dental health”or”occupational accidents“. Only two studies [36, 37] analyzed health topics covered by posts in social media (Twitter and Instagram).

Compared to the study by Paul and Dredze [36] on health topics on Twitter, we identified similar themes and/or topics within the sGHW such as “cancer & serious illness”, “injuries & pain”, “diet & exercise” and “family”. Muralidhara and Paul [37] explored health topics on Instagram and discovered ten broad categories. Compared to their work, we were able to identify similar topics such as “acute illness”, “alternative medicine”, “chronic illness and pain”, “mental health”, “diet” as well as “substance use”.

In contrast to the studies by Paul and Dredze [36] and Muralidhara and Paul [37], we focused on the German language and the sGHW rather than on social media. In addition, contrary to [38–42], we explored general health topics within the sGHW rather than focusing on one certain (health-related) discipline.

We found topic representations of the most common diseases in the D-A-CH region such as “Oncology” (T5), “Chronic Illness” (T21), “Pain” (T26), “Heart diseases (T28)”, “Mental Illness” (T31), “Pandemic & Vaccination” (T35), “Respiratory & pulmonary diseases” (T42) and “Neurological diseases” (T46) [79]. Interestingly, T35 includes terms such as “covid” and “vaccination” referring to the current pandemic situation although the European outbreak started in the last months of our web crawl. In addition, (healthy) food (T12, T39, T41), food intolerance (T2) as well as food production (T14, T43, T44, T47) seem to play an important role within the sGHW.

#### Conclusions and further research

In this study, a system was presented which computes the readability and vocabulary difficulty of health-related web pages gathered by a focused web crawler in a fully-automated way. We showed, that a graph representation of the sGHW can be extracted during the data collection phase, which can then be used to compute a ranking of the top 1000 web sites for the ccTLDs “.de”, “.at”, and “.ch”. In addition, we demonstrated that LDA can be used to explore the collected dataset. In total, we were able to identify 50 topics, which were summarized into 11 themes.

Our results indicate that the readability within the sGHW is low. For this reason, publishing organizations and authors should reevaluate existing text material and reduce sentence complexity. However, our findings suggest that the use of vocabulary often suits the target audience but could be improved. Therefore, we recommend the use of both sentence dimension and vocabulary dimension as supportive measures to ensure and provide understandable online health information. Therefore, content providers should be supported by proper tooling during text production: I.e., one could envision a cloud service where health content providers could check their health-related web content automatically for readability and vocabulary difficulty. In addition, users should be supported by proper browser-based tooling (i.e., browser extensions such as [29]) to identify easy-to-read content but also to get an indication of the quality of the related content.

In future work, the authors intend to extend their analyses to identify trustworthy health information web sites. To do so, we plan to combine the DISCERN instrument [86] with crowd-sourcing approaches. Using these insights and with the acquired data available, an implementation and evaluation of a trustworthy health-specific search engine for information seeking citizens will be possible.

## Supporting information

S1 Appendix Overview of information provider categories. (DOCX)

S2 Appendix Instructions for volunteers written in German and word clouds to name. (XLSX)

S3 Appendix Top-ranked 1000 web sites for each ccTLD, their linguistic characteristics and the related text difficulty. (XLSX)

S4 Appendix Class distribution for FRE, WSTF and L for each web site category. (DOCX)

S5 Appendix Topics inferred by LDA in word clouds representation. (ZIP)

## Abbreviations

API: Application Programming Interface
ASL: Average Sentence Length
ASW: Average Number of Syllables per Word
ccTLD: country-code top level domain
FKG: Flesch-Kincaid Grade
FRE: Flesch Reading Ease Scale
GHW: German Health Web
L: vocabulary measure
LDA: Latent Dirichlet Allocation
MS: Words with three or More Syllables
NLP: Natural Language Processing
PA: Percent Agreement
PCC: Pearson correlation coefficient
SEO: Search Engine Optimization
sGHW: Sampled German Health Web
SMOG: Simple Measure of Gobbledygook score
SVM: Support Vector Machine
TLD: top level domain
WSTF: 4^th^ Vienna Formula (German: Wiener SachTextFormel)
