# Supplementary material for: Readability and topics of the German Health Web: Exploratory study and text analysis
Source: PLoS One. 2023 Feb 10;18(2):e0281582. doi: 10.1371/journal.pone.0281582 (PMC9916670; doi:10.1371/journal.pone.0281582)
Supplement: S1 Appendix — (DOCX) [file pone.0281582.s001.docx]

# Supporting Information 1

## Overview of Information Provider Types

### Government or Public (Health) Institution (GPH)

### This category includes web sites provided by government agencies or government-related organizations or have an educational or research related background. These include, for example, the Robert Koch Institute (ww.rki.de), web sites of ministries (www.bundesgesundheitsministerium.de) or state or state-recognized universities (www.uzh.ch). In addition, important actors in the field of research such as the German Cancer Research Center (www.dkfz.de) or the Fraunhofer Society (fraunhofer.de) are included in this category.

### Non-Profit Organization (NPO)

This category includes web sites of non-profit associations or foundations, eg German Cancer Aid (www.krebshilfe.de) or the German Nutrition Society (www.dge.de). In addition, self-help groups such as www.psoriasis-netz.de are included in this category.

### Private Organization or Individual Person (PO)

This category includes web sites of private organizations or individual persons, eg web sites of medical doctors, dental laboratories or health-related services such as physiotherapy. In addition, this category also contains web sites related to well-being and health prevention. For example, a furniture store explaining healthy sitting positions would also be included in this category.

### Mainstream or Local News (M)

This category includes web sites which are operated by news agencies or related organisations. Examples include the web site of newspapers such as “Der Spiegel” (www.spiegel.de) or “Die Welt” (www.welt.de). In addition, public broadcasting companies such as the Northern German Broadcasting (www.ndr.de) are also included in this category.

### Pharmaceutical Company (PC)

This category includes web sites of pharmaceutical companies or with pharmaceutical associations.

### Personal Blog (PB)

This category includes personal web blogs of individual persons, eg blogging about one's illness(es) or recommending treatment or therapy options.

### Social Network (SN)

This category includes social networks such as Twitter, Facebook or Reddit.

### Other (O)

This category includes search engines and other types of web sites. It is also assigned, if a given web site is not reachable (eg. HTTP Status Code 404 or 500) or does not contain any content.
