# Supplementary material for: Readability and topics of the German Health Web: Exploratory study and text analysis
Source: PLoS One. 2023 Feb 10;18(2):e0281582. doi: 10.1371/journal.pone.0281582 (PMC9916670; doi:10.1371/journal.pone.0281582)
Supplement: S4 Appendix — (DOCX) [file pone.0281582.s004.docx]

Table 1. Distribution of readability values on the Flesch Reading Ease scale with difficulty mapping per web site category for “.de”, “.at” and “.ch”. V: very easy, E: easy, M: moderate, D: difficult, VD: very difficult, N/A: computation not possible, -: not applicable. Information provider types: GPH: Government, Public Institution or Public Health, NPO: Non-Profit Organization, Private Organization: PO, Mainstream or Local News: M, Pharmaceutical Company: PC, Other: O

|  | **Total (%)** | | | **VE (%)** | | | **E (%)** | | | **M (%)** | | | **D (%)** | | | **VD (%)** | | | **N/A (%)** | | |
| --- | --- | --- | --- | --- | --- | --- | --- | --- | --- | --- | --- | --- | --- | --- | --- | --- | --- | --- | --- | --- | --- |
| **ccTLD** | **de** | **at** | **ch** | **de** | **at** | **ch** | **de** | **at** | **ch** | **de** | **at** | **ch** | **de** | **at** | **ch** | **de** | **at** | **ch** | **de** | **at** | **ch** |
| **Category** |  |  |  |  |  |  |  |  |  |  |  |  |  |  |  |  |  |  |  |  |  |
| GPH | 214 (100) | 145 (100) | 196 (100) | - | - | - | - | - | 1 (0.5) | - | - | - | 17 (7.9) | 18 (12.4) | 40 (20.4) | 180 (84.1) | 116 (80.0) | 133 (67.9) | 17 (7.9) | 11 (7.6) | 22 (11.2) |
| NPO | 237 (100) | 147 (100) | 157 (100) | - | - | - | - | - | - | - | - | 1 (0.6) | 46 (19.4) | 24 (16.3) | 45 (28.7) | 164 (69.2) | 102 (69.4) | 88 (56.1) | 27 (11.4) | 21 (143) | 23 (14.7) |
| PO | 435 (100) | 603 (100) | 583 (100) | - | - | - | - | - | - | - | - | 2 (0.3) | 156 (35.9) | 205 (34.0) | 216 (37.2) | 243 (55.9) | 339 (56.2) | 318 (54.5) | 36 (8.276) | 59 (9.784) | 47 (8.1) |
| M | 62 (100) | 40 (100) | 20 (100) | - | - | - | - | - | - | - | - | - | 48 (77) | 31 (78) | 20 (100) | 10 (16) | 9 (23) | - | 4 (7) | - | - |
| PC | 39 (100) | 46 (100) | 31 (100) | - | - | - | - | - | - | - | - | - | 11 (28) | 24 (52) | 12 (39) | 24 (62) | 22 (48) | 18 (58) | 4 (10) | - | 1 (3) |
| PB | 8 (100) | 12 (100) | 7 (100) | - | - | - | - | - | - | - | - | - | 3 (38) | 3 (25) | 6 (86) | 2 (25) | 8 (67) | 1 (14) | 3 (38) | 1 (8) | - |
| O | 5 (100) | 7 (100) | 6 (100) | - | - | - | - | - | - | - | - | - | 2 (40) | 2 (29) | 1 (17) | 2 (40) | 4 (57) | 3 (50) | 1 (20) | 1 (14) | 2 (33) |
| Total | 1000 (100) | 1000 (100) | 1000 (100) | - | - | - | - | - | 1 (0.100) | - | - | 3 (0.300) | 283 (28.30) | 307 (30.70) | 340 (34.00) | 625 (62.50) | 600 (60.00) | 561 (56.10) | 92 (9.20) | 93 (9.30) | 95 (9.50) |

Table 2. Distribution of readability values of the 4^th^Vienna formula scale with difficulty mapping per web site category for “.de”, “.at” and “.ch”. V: very easy, E: easy, M: moderate, D: difficult, VD: very difficult, N/A: computation not possible, -: not applicable. Information provider types: GPH: Government, Public Institution or Public Health, NPO: Non-Profit Organization, Private Organization: PO, Mainstream or Local News: M, Pharmaceutical Company: PC, Other: O

|  | **Total (%)** | | | **VE (%)** | | | **E (%)** | | | **M (%)** | | | **D (%)** | | | **VD (%)** | | | **N/A (%)** | | |
| --- | --- | --- | --- | --- | --- | --- | --- | --- | --- | --- | --- | --- | --- | --- | --- | --- | --- | --- | --- | --- | --- |
| **ccTLD** | **de** | **at** | **ch** | **de** | **at** | **ch** | **de** | **at** | **ch** | **de** | **at** | **ch** | **de** | **at** | **ch** | **de** | **at** | **ch** | **de** | **at** | **ch** |
| **Category** |  |  |  |  |  |  |  |  |  |  |  |  |  |  |  |  |  |  |  |  |  |
| GPH | 214 (100) | 145 (100) | 196 (100) | - | - | - | - | - | 3 (1.5) | 2 (0.9) | 2 (1.4) | 6 (3.1) | 113 (52.8) | 80 (55.2) | 131 (66.8) | 82 (38.2) | 52 (35.9) | 34 (17.4) | 17 (7.9) | 11 (7.6) | 22 (11.2) |
| NPO | 237 (100) | 147 (100) | 157 (100) | - | - | - | - | - | 3 (1.9) | 3 (1.3) | 2 (14) | 3 (1.9) | 164 (69.2) | 85 (57.8) | 109 (69.4) | 43 (18.1) | 39 (26.5) | 19 (12.1) | 27 (11.4) | 21 (14.2) | 23 (14.7) |
| PO | 435 (100) | 603 (100) | 583 (100) | 1 (0.2) | - | - | 2 (0.5) | 5 (0.8) | 3 (0.5) | 29 (6.7) | 43 (7.1) | 45 (7.7) | 271 (62.3) | 386 (64.0) | 383 (65.7) | 96 (22.1) | 110 (18.2) | 105 (18.0) | 36 (8.3) | 59 (9.8) | 47 (8.1) |
| M | 62 (100) | 40 (100) | 20 (100) | - | - | - | - | - | - | 7 (11) | 4 (10) | 4 (20) | 49 (79) | 36 (90) | 16 (80) | 2 (3) | - | - | 4 (6) | - | - |
| PC | 39 (100) | 46 (100) | 31 (100) | - | - | - | - | - | - | 1 (3) | 7 (15) | 2 (7) | 25 (64) | 29 (63) | 24 (77) | 9 (23) | 10 (22) | 4 (13) | 4 (10) | - | 1 (3) |
| PB | 8 (100) | 12 (100) | 7 (100) | - | - | - | - | - | - | - | - | 2 (29) | 4 (50) | 10 (83) | 5 (71) | 1 (13) | 1 (8) | - | 3 (38) | 1 (8) | - |
| O | 5 (100) | 7 (100) | 6 (100) | - | - | - | - | - | - | 1 (20) | - | 1 (17) | 2 (40) | 5 (71) | 2 (33) | 1 (20) | 1 (14) | 1 (17) | 1 (20) | 1 (14) | 2 (33) |
| Total | 1000 (100) | 1000 (100) | 1000 (100) | 1 (0.10) | - | - | 2 (0.20) | 5 (0.50) | 9 (0.90) | 43 (4.30) | 58 (5.80) | 63 (6.30) | 628 (62.80) | 631 (63.10) | 670 (67.00) | 234 (23.40) | 213 (21.30) | 163 (16.30) | 92 (9.20) | 93 (9.30) | 95 (9.50) |

Table 3. Distribution of readability values on the SVM classification with difficulty mapping per web site category for “.de”, “.at” and “.ch”. V: very easy, E: easy, M: moderate, D: difficult, VD: very difficult, N/A: computation not possible, -: not applicable. Information provider types: GPH: Government, Public Institution or Public Health, NPO: Non-Profit Organization, Private Organization: PO, Mainstream or Local News: M, Pharmaceutical Company: PC, Other: O

|  | **Total (%)** | | | **VE (%)** | | | **E (%)** | | | **M (%)** | | | **D (%)** | | | **VD (%)** | | | **N/A (%)** | | |
| --- | --- | --- | --- | --- | --- | --- | --- | --- | --- | --- | --- | --- | --- | --- | --- | --- | --- | --- | --- | --- | --- |
| **ccTLD** | **de** | **at** | **ch** | **de** | **at** | **ch** | **de** | **at** | **ch** | **de** | **at** | **ch** | **de** | **at** | **ch** | **de** | **at** | **ch** | **de** | **at** | **ch** |
| **Category** |  |  |  |  |  |  |  |  |  |  |  |  |  |  |  |  |  |  |  |  |  |
| GPH | 214 (100) | 145 (100) | 196 (100) | 5 (2.3) | - | 6 (3.1) | 14 (6.5) | 12 (8.3) | 25 (12.8) | 14 (6.5) | 14 (9.7) | 25 (12.8) | 90 (42.1) | 77 (53.1) | 83 (42.3) | 74 (34.6) | 31 (21.4) | 35 (17.9) | 17 (7.9) | 11 (7.6) | 22 (11.2) |
| NPO | 237 (100) | 147 (100) | 157 (100) | 4 (1.7) | 5 (3.4) | 12 (7.6) | 21 (8.9) | 16 (10.9) | 23 (14.7) | 20 (8.4) | 9 (6.1) | 18 (11.5) | 96 (40.5) | 61 (41.5) | 51 (32.5) | 69 (29.1) | 35 (23.8) | 30 (19.1) | 27 (11.4) | 21 (143) | 23 (14.70) |
| PO | 435 (100) | 603 (100) | 583 (100) | 58 (13.3) | 85 (14.1) | 56 (9.6) | 77 (17.7) | 141 (23.4) | 150 (25.7) | 59 (13.6) | 76 (12.6) | 67 (11.5) | 125 (28.7) | 150 (24.9) | 159 (27.3) | 79 (18.2) | 92 (15.3) | 104 (17.8) | 37 (8.5) | 59 (9.8) | 47 (8.1) |
| M | 62 (100) | 40 (100) | 20 (100) | 8 (13) | - | 1 (5) | 21 (34) | 20 (50) | 6 (30) | 18 (29) | 8 (20) | 5 (25) | 9 (15) | 10 (25) | 8 (40) | 2 (3) | 2 (5) | - | 4 (6) | - | - |
| PC | 39 (100) | 46 (100) | 31 (100) | 7 (18) | 11 (24) | 1 (3) | 7 (18) | 14 (30) | 8 (26) | 4 (10) | 4 (9) | 5 (16) | 14 (36) | 15 (33) | 11 (36) | 3 (8) | 2 (4) | 5 (16) | 4 (10) | - | 1 (3) |
| PB | 8 (100) | 12 (100) | 7 (100) | - | - | - | 1 (13) | 3 (25) | 4 (57) | 1 (13) | - | 1 (14) | 1 (13) | 7 (58) | 2 (29) | 2 (25) | 1 (8) | - | 3 (38) | 1 (8) | - |
| O | 5 (100) | 7 (100) | 6 (100) | 1 (20) | - | 1 (17) | 2 (40) | 1 (14) | 2 (33) | - | - | - | - | 5 (71) | - | 1 (20) | - | 1 (17) | 1 (20) | 1 (14) | 2 (33) |
| Total | 1000 (100) | 1000 (100) | 1000 (100) | 83 (8.30) | 101 (10.10) | 77 (7.70) | 143 (14.30) | 207 (20.70) | 218 (21.80) | 116 (11.60) | 111 (11.10) | 121 (12.10) | 335 (33.50) | 325 (32.50) | 314 (31.40) | 230 (23.00) | 163 (16.30) | 175 (17.50) | 93 (9.30) | 93 (9.30) | 95 (9.50) |
