# Supplementary figures and images for: Readability and topics of the German Health Web: Exploratory study and text analysis
Source: PLoS One. 2023 Feb 10;18(2):e0281582. doi: 10.1371/journal.pone.0281582 (PMC9916670; doi:10.1371/journal.pone.0281582)

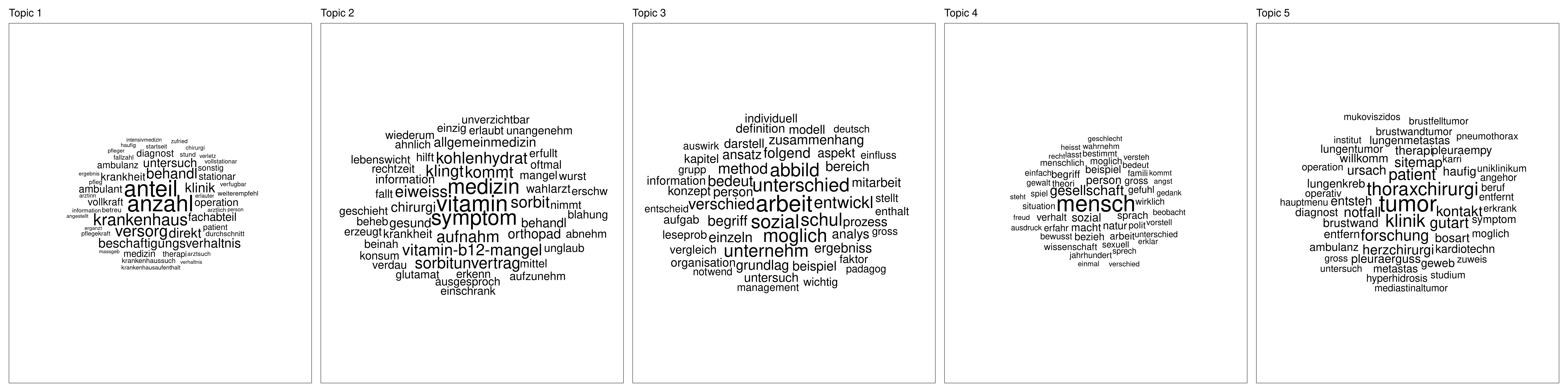

Supplement: S5 Appendix — (ZIP) [file pone.0281582.s005.zip › 1-5.png]

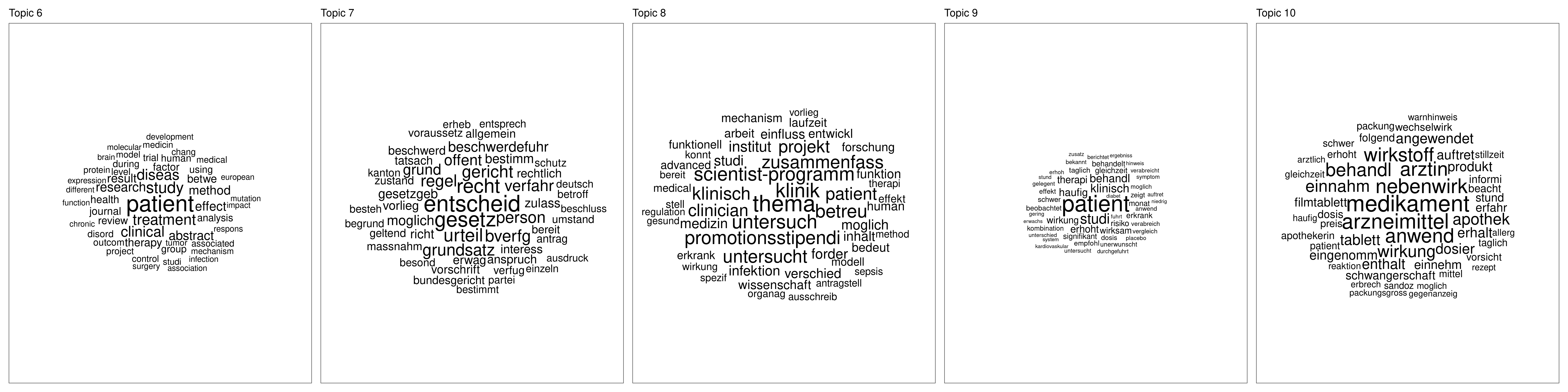

Supplement: S5 Appendix — (ZIP) [file pone.0281582.s005.zip › 6-10.png]

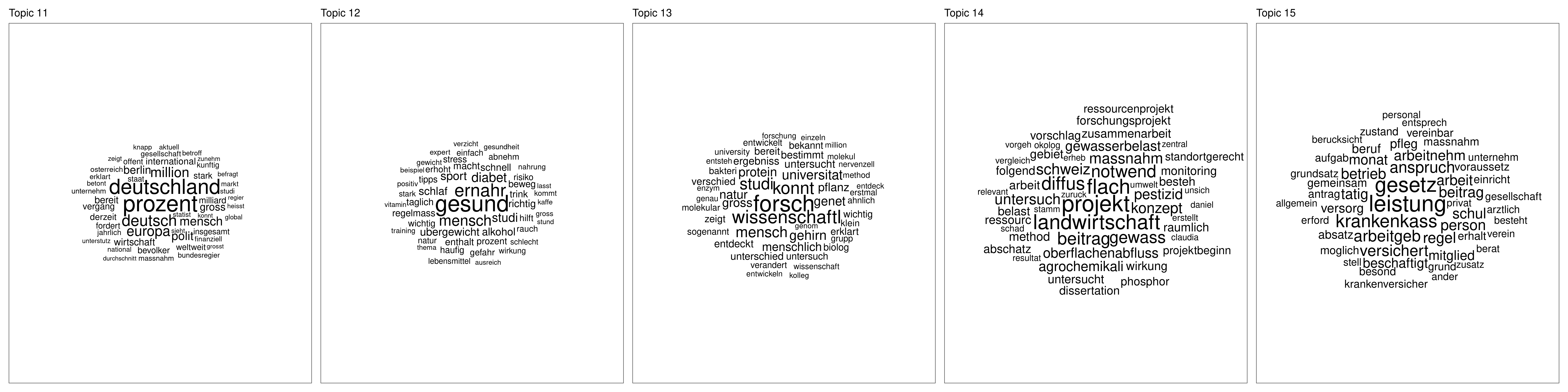

Supplement: S5 Appendix — (ZIP) [file pone.0281582.s005.zip › 11-15.png]

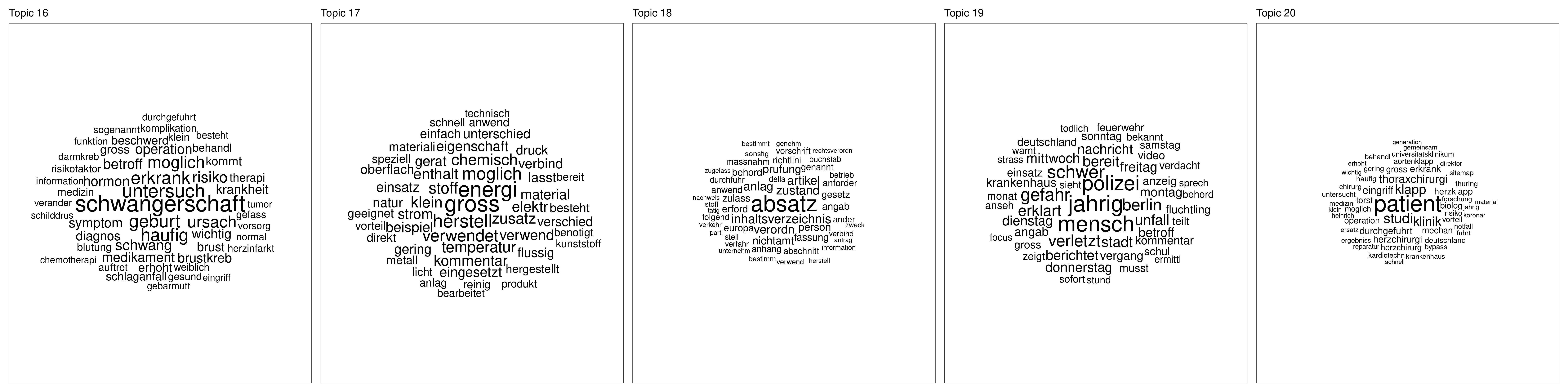

Supplement: S5 Appendix — (ZIP) [file pone.0281582.s005.zip › 16-20.png]

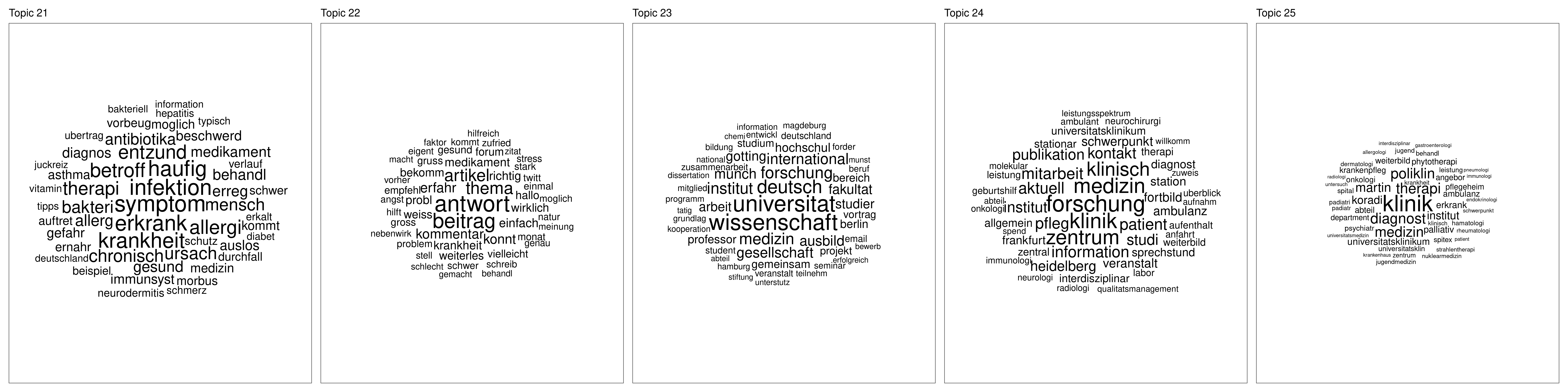

Supplement: S5 Appendix — (ZIP) [file pone.0281582.s005.zip › 21-25.png]

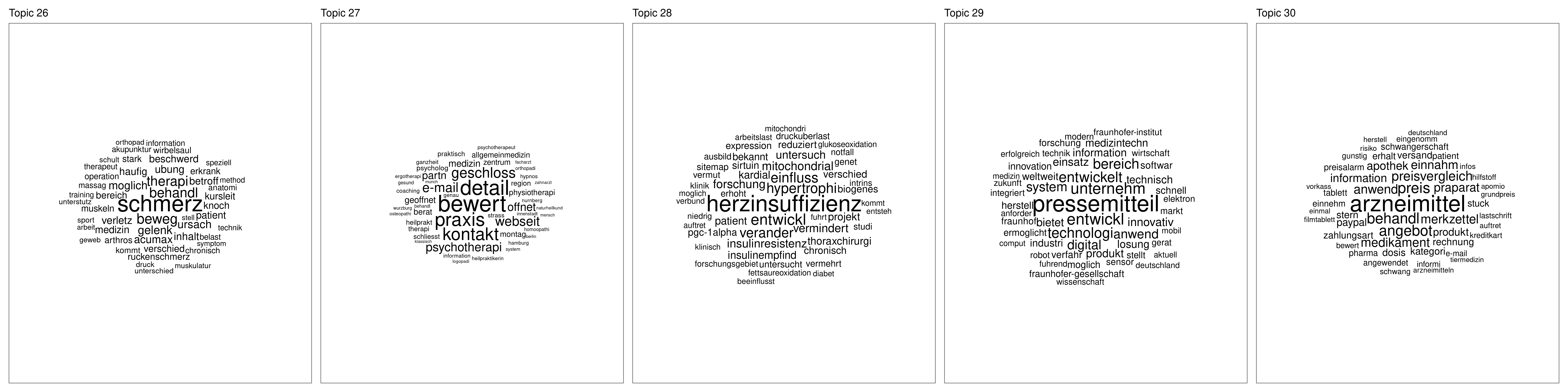

Supplement: S5 Appendix — (ZIP) [file pone.0281582.s005.zip › 26-30.png]

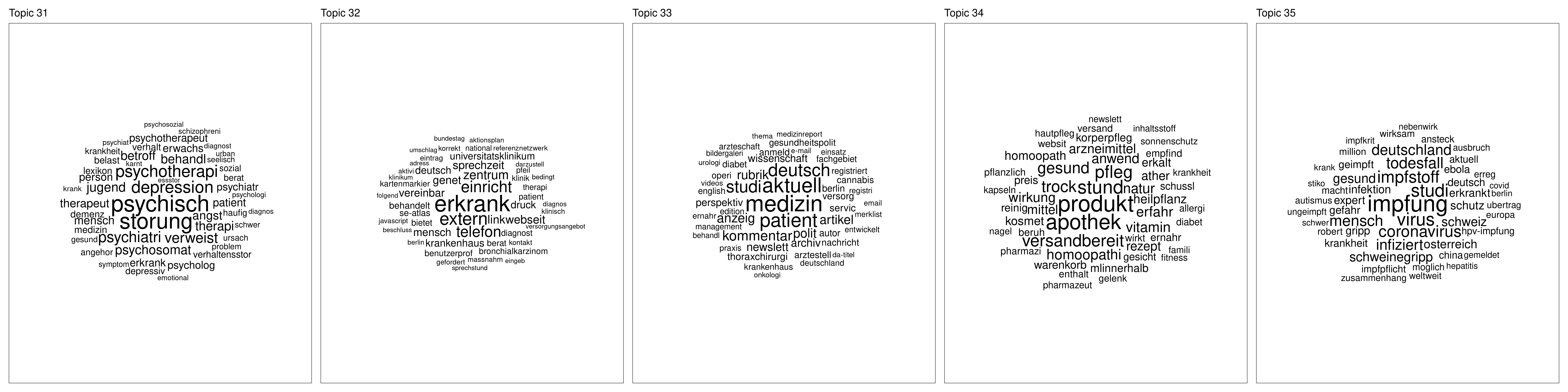

Supplement: S5 Appendix — (ZIP) [file pone.0281582.s005.zip › 31-35.png]

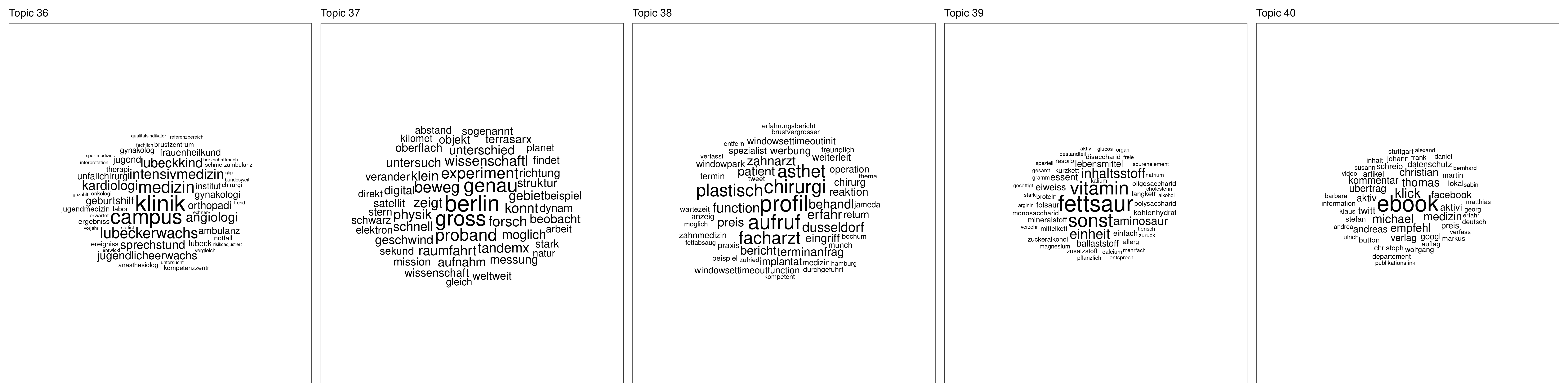

Supplement: S5 Appendix — (ZIP) [file pone.0281582.s005.zip › 36-40.png]

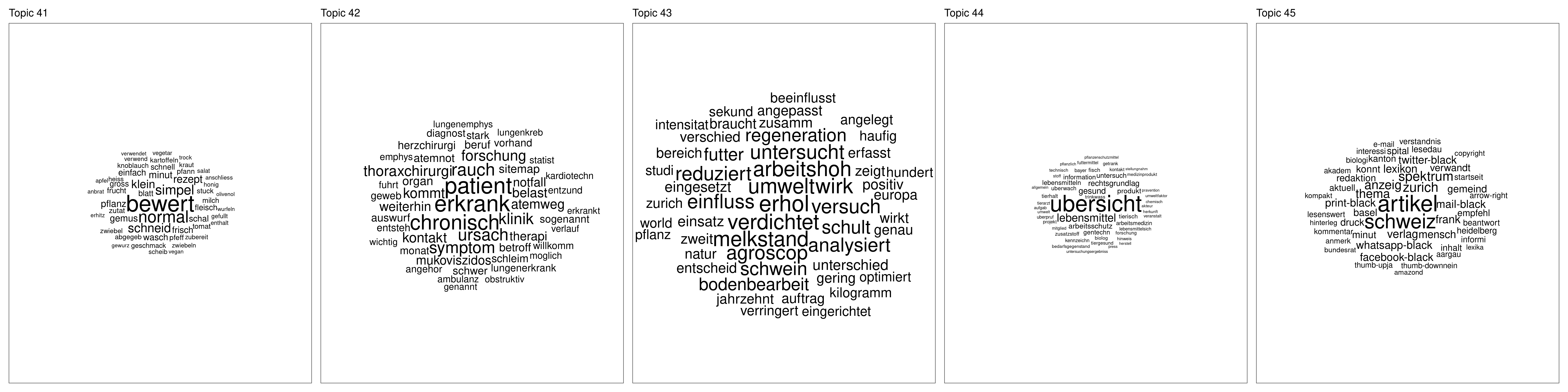

Supplement: S5 Appendix — (ZIP) [file pone.0281582.s005.zip › 41-45.png]

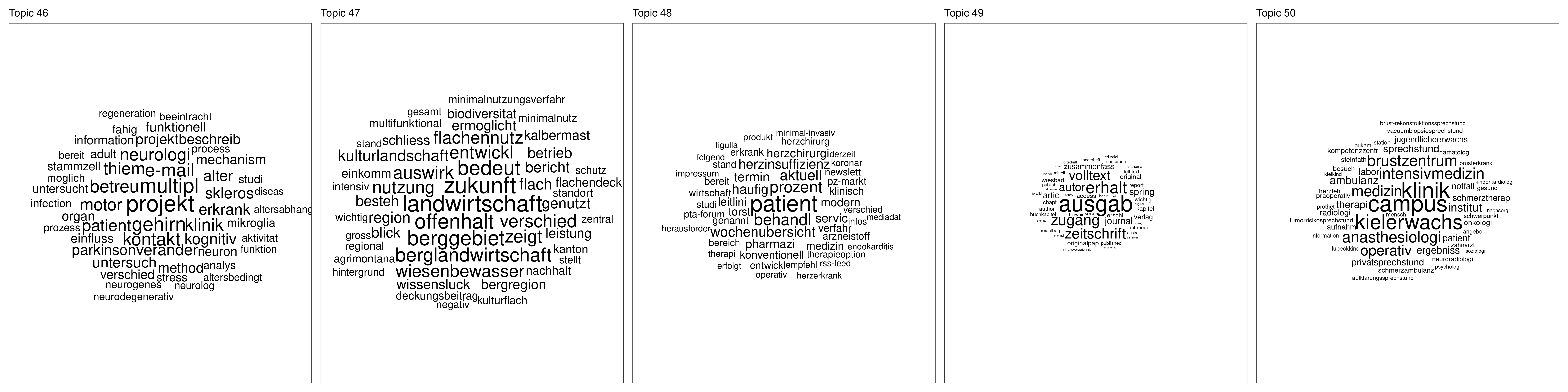

Supplement: S5 Appendix — (ZIP) [file pone.0281582.s005.zip › 46-50.png]
